# Supplementary material for: Field theory for recurrent mobility
Source: Nat Commun. 2019 Aug 29;10:3895. doi: 10.1038/s41467-019-11841-2 (PMC6715718; doi:10.1038/s41467-019-11841-2)
Supplement: Supplementary file 1 — Supplementary Information [file 41467_2019_11841_MOESM1_ESM.pdf]

Supplementary Information
Field Theory for recurrent mobility

M. Mazzoli, A. Molas, A. Bassolas, M. Lenormand, P. Colet & J.J. Ramasco

Contents

Supplementary Note 1: Calculation of fluxes 4

Supplementary Note 2: Validation with census data 4

Supplementary Note 3: Fitting the gravity model parameters 11

Supplementary Note 4: Analytical approaches 12

Supplementary Note 5: The gravity model with a power-law deterrence function 16

Supplementary Note 6: Gravity vs Radiation: model comparison 19

Supplementary Note 7: Further cities with Twitter data 22

Supplementary Note 8: Further equipotential curves 31

Supplementary Note 9: Fluctuations in the empirical curl 32

Supplementary Note 10: Trip distance distribution 32

Supplementary Note 11: Angle distribution 33

Supplementary Note 12: Collection of geolocated Twitter data 35

Supplementary Note 13: An alternative definition of the home-work flows 36

Supplementary Figures

List of Figures

1	Gauss Theorem verification with census data	5
2	Empirical vector field analysis with census data in London	5
3	Potential validation with census data in London	6
4	Flux analysis with census data in London	6
5	Empirical vector field analysis with census data in Manchester	7
6	Potential comparison with census data in Manchester	7
7	Flux analysis with census data in Manchester	7
8	Empirical vector field analysis with census data in Paris	8
9	Potential comparison with census data in Paris	9
10	Flux analysis with census data in Paris	9
11	Mass distributions	10
12	Redistributing census area population into grid cells	10
13	Fitted d_0 distributions boxplot for various thresholds	11
14	Parameterization scheme	13
15	Gauss Theorem for the gravity model	13
16	Analytical fits of the maxima	14
17	Flux-curves collapse	14
18	Semi-analytical fluxes in London	15
19	Flux analysis in London with a power-law gravity model $\gamma = 1$	17
20	Flux analysis in London with a power-law gravity model $\gamma = 2$	17
21	Potential analysis with a power-law gravity model	18
22	Power-law flux curves collapse	18
23	Radiation Model predicted vector field in London	19
24	Model comparison in London	20
25	Model comparison in Los Angeles	21
26	Model comparison in Tokyo	21
27	Vector field comparison in Manchester	22
28	Potential comparison in Manchester	22
29	Flux comparison in Manchester	23
30	Vector field comparison in Paris	24
31	Potential comparison in Paris	24
32	Flux comparison in Paris	25
33	Vector field comparison in Los Angeles	26
34	Potential comparison in Los Angeles	26
35	Flux comparison in Los Angeles	27
36	Vector field comparison in Tokyo	28
37	Potential comparison in Tokyo	28
38	Flux comparison in Tokyo	29
39	Vector field comparison in Rio de Janeiro	30
40	Potential comparison in Rio de Janeiro	30
41	Flux comparison in Rio de Janeiro	31
42	Empirical equipotential lines	32
43	Curl distribution in London	32
44	Trip distance distributions	33
45	Vector-field angle distributions	33
46	Vector field angle complementary cumulative distribution	33
47	Vector field angle comparison	34
48	Angle Delta-map	34
49	Angle null model Delta-map	35
50	Δ distribution comparison	35
51	Inverting the flows in Paris	36

Supplementary Tables

List of Tables

1 List of central coordinates for each city. 4
2 List of R_p^2 correlation values for each city with circle and square surfaces. 4
3 List of d_0 fitted parameters and Twitter users per city. 11
4 List of k fitted parameters per city. 12

Supplementary Note 1: Calculation of fluxes

The centers of the cities for the flux calculations used to check Gauss Theorem have been taken at locations shown in Table 1.

City	*Latitude*	*Longitude*
London	51.509	-0.118
Manchester	53.483	-2.244
Paris	48.864	2.349
Tokyo	35.652	139.839
Rio de Janeiro	-22.970	-43.182
Los Angeles	34.052	-118.243

Supplementary Table 1: List of central coordinates for each city.

The R_p^2 for the comparison between fluxes calculated as surface and volume integrals with Twitter data for all the cities in the Table 2.

City	*Circle*	*Square*
London	0.97	0.89
Manchester	0.85	0.66
Paris	0.97	0.80
Tokyo	0.95	0.87
Rio de Janeiro	0.96	0.70
Los Angeles	0.93	0.82

Supplementary Table 2: List of R_p^2 correlation values for each city with circle and square surfaces.

Supplementary Note 2: Validation with census data

Divergence theorem

In the main paper, we have shown that the Gauss Divergence Theorem holds with Twitter data. Here we show that it also holds if the analysis is performed with census data.

Supplementary Figure 1 displays the flux measured for circles and squares centered in London and Paris as the Figure 2 of the main manuscript. The flux calculated with the surface integration and the volume integral of the divergence of $\vec{\mathbf{W}}$ display in both cities (Paris and London) a very good agreement, as the high values R_p^2 demonstrate.

London

As can be seen, even with London census data the modulus of the curl of the vector field W defined by the commuters of the city is negligible if compared to the typical modulus values of the same vector field in every cell. Given these results, we can define a potential from the real data as well.

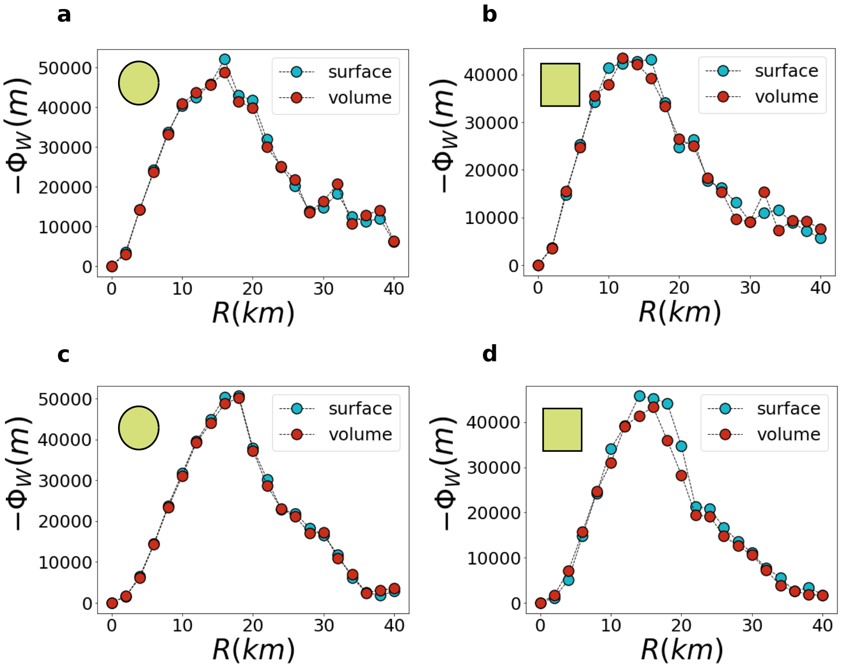

Supplementary Figure 1: **Gauss Theorem verification with census data. a** London, surface flux integral, $R_p^2 = 0.98$. **b** London, volume divergence integral, $R_p^2 = 0.98$. **c** Paris, surface flux integral, $R_p^2 = 1.00$. **d** Paris, volume divergence integral, $R_p^2 = 0.98$.

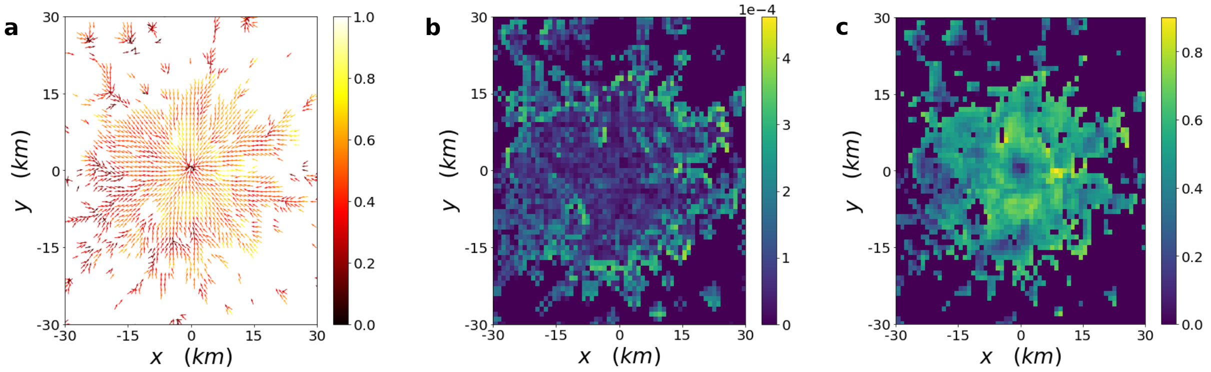

Supplementary Figure 2: **Empirical vector field analysis. a** London census data vector field W. **b** London census data vector field W curl modulus in m^{-1}. **c** London census data modulus of vector field W.

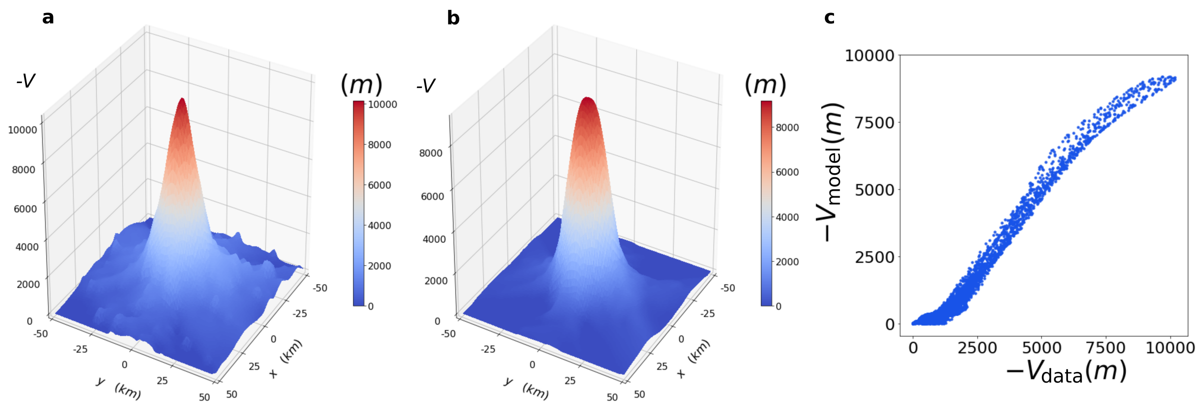

Supplementary Figure 3: **Potential validation.** **a** London census data empirical potential. **b** London census data Gravity model with an exponential deterrence function predicted potential. **c** London potentials correlation plot. $R_p^2 = 0.97$

The gravity model predicted potential is highly correlated to the empirical one observed, as shown in Figure 3c. The fluxes of commuting workers measured in London using the census data is shown in Supplementary Figure 4.

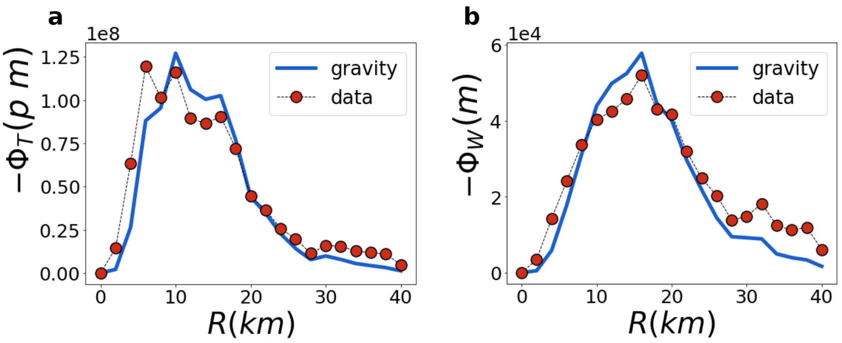

Supplementary Figure 4: **Fluxes analysis.** **a** London census data real entering flux of T. $R_p^2 = 0.91$. **b** London census data real flux of vector field W. $R_p^2 = 0.96$.

Manchester-Liverpool

The vectors easily depict the centers of attraction represented by the cities of Liverpool, Manchester, Sheffield, Leeds and many more smaller towns.

The curl of the vector field is almost zero and negligible with respect to the modulus of the vectors in all northern England.

The gravity model predicted potential gives a good approximation of the empirical computed potential. Both potentials also show the strongest centers of attraction in the area considered.

The high correlation highlights the good approximation given by the gravity model to the empirical fluxes, at great distances as well, even if with some more noise with respect to larger cities. This noise originates by the presence of small agglomerates around the whole conurbation area which are subject to fluctuations, differently from highly crowded zones.

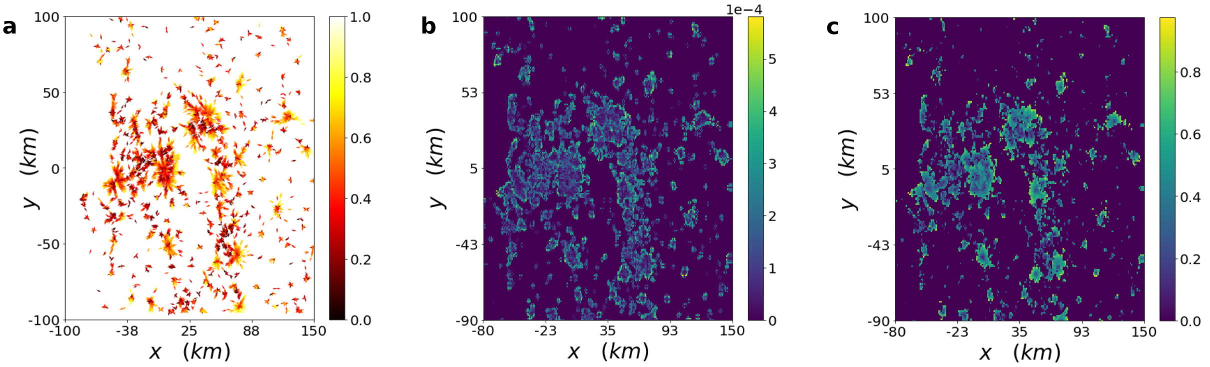

Supplementary Figure 5: **Empirical vector field analysis.** **a** Manchester census data vector field W. **b** Manchester census data vector field W curl modulus in m^{-1}. **c** Manchester census data modulus of vector field W.

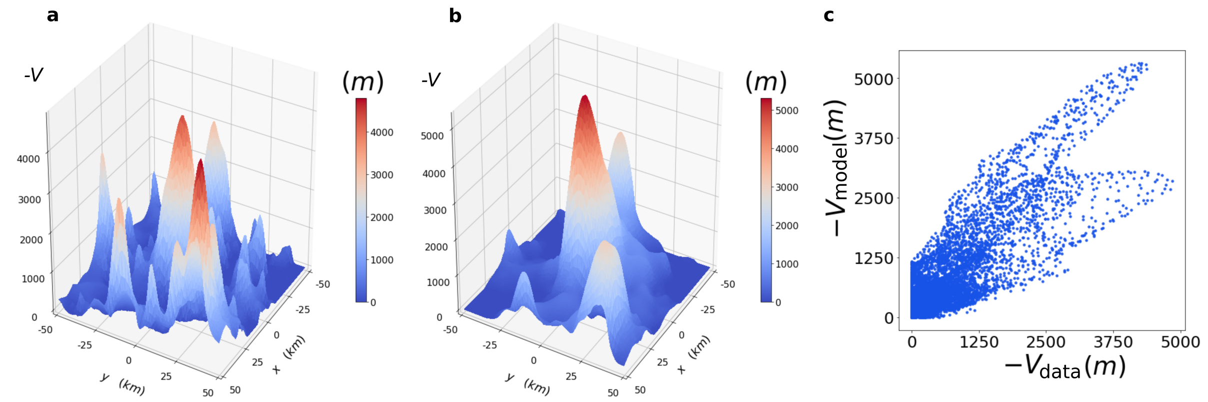

Supplementary Figure 6: **Potential comparison.** **a** Manchester census data empirical potential. **b** Manchester census data Gravity model predicted potential. **c** Manchester potentials correlation plot. $R_p^2 = 0.69$

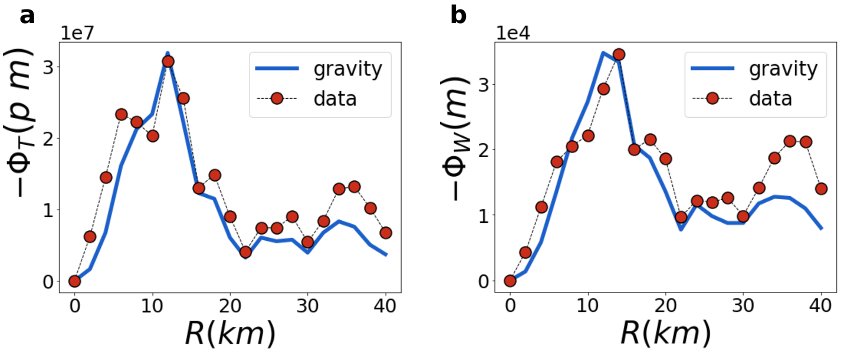

Supplementary Figure 7: **Fluxes analysis.** **a** ManLiv census data real entering flux of T. $R_p^2 = 0.95$ **b** ManLiv census data real flux of vector field W. $R_p^2 = 0.63$.

Paris

We extracted the data of commuting flows in France from the public French census of 2011. Using O-D matrices we reproduced the same analyses as in the main paper with Twitter for the city of Paris.

Looking at Supplementary Figure 8a we can easily spot the core of the city of Paris. From Supplementary Figure 8b we see as the rotational is almost null in all the region corresponding to Paris, except for some peripheral zones, which still are negligible in comparison with the modulus in Supplementary Figure 8c. This allows us to define a potential as we did above for the Twitter data.

As can be seen in Supplementary Figure 9, the model predicted potential reproduces the observed potential built from the commuting vectors. The agreement in shown in Supplementary Figure 9c.

Figure 10 shows the comparison between the real data computed flux and the flux we get through the gravity model.

We conclude that the gravity model with an exponential deterrence function with d_0 in Table S1 works well with census data from France.

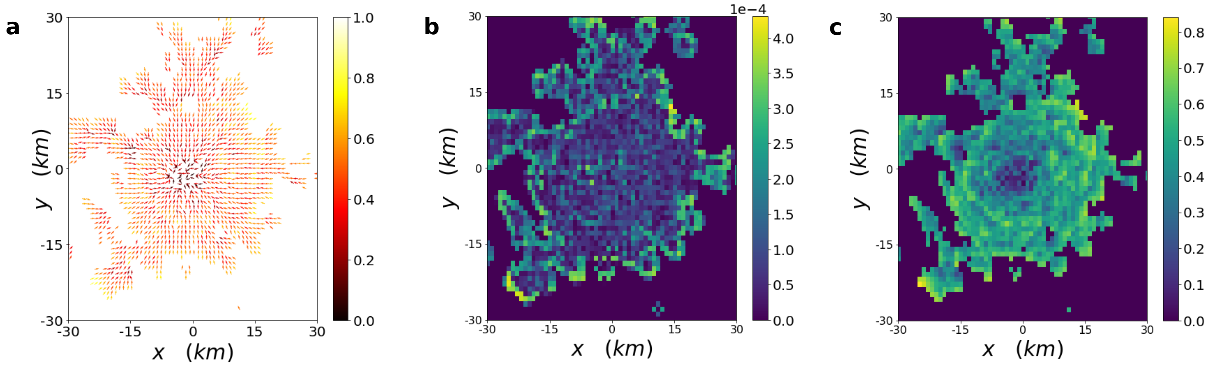

Supplementary Figure 8: **Empirical vector field analysis.** **a** Paris census data vector field W. **b** Paris census data vector field W curl modulus in m^{-1}. **c** Paris census data modulus of vector field W.

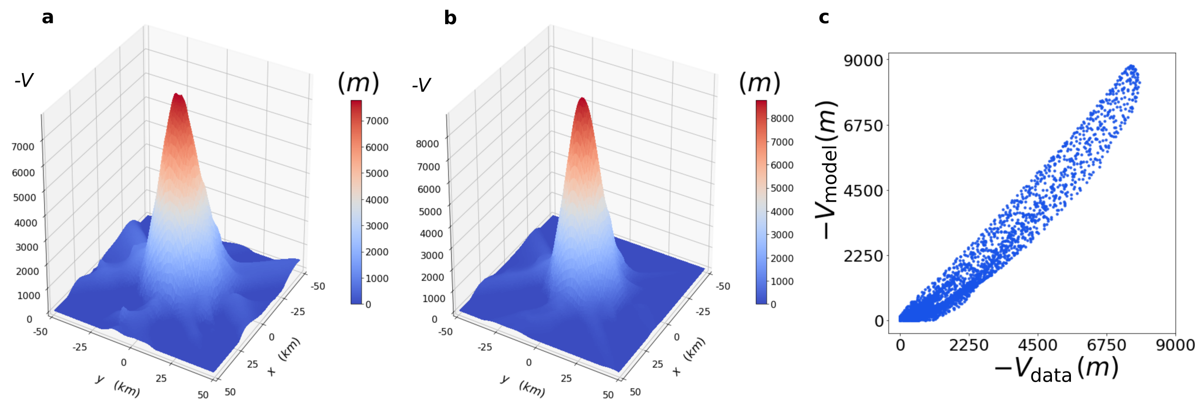

Supplementary Figure 9: **Potential comparison.** **a** Paris census data empirical potential. **b** Paris census data Gravity model predicted potential. **c** Paris potentials correlation plot. $R_p^2 = 0.94$

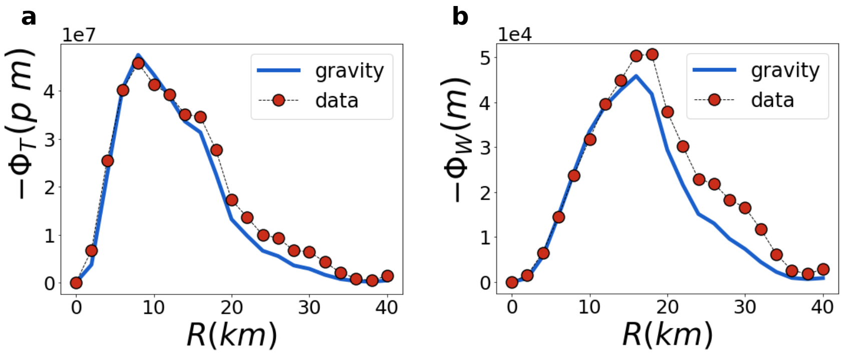

Supplementary Figure 10: **Fluxes analysis.** **a** Paris commuters entering flux of T, $R_p^2 = 0.98$. **b** Paris commuters entering flux of W, $R_p^2 = 0.95$.

Mapping census data to a grid and filters

To avoid considering noisy fluctuations in cells in terms of home places and work places, we filtered our data. In the case of Twitter data, all cells with less than 5 residents or workers have been filtered out. In the case of census data, we used as threshold the 15% of the maximum value of residents per cell. In Supplementary Figure 11, we can see the population distribution in various cities.

Census data is produced in convenient geographical areas that are heterogeneous in size and in spatial distribution. In order to map the results in a grid, we need to develop a procedure taking into account the overlap between census cells and grid cells. We assume that both the population and mobility can be distributed according to the overlapping area between cells. We need to calculate the spatial overlap of census area A_c and grid cell A_i and assign to A_i a fraction of the population m_c and the mobility of A_c given by the ratio between $|A_c \cap A_i|/|A_c|$, where $|A_{c/i}|$ is the surface of $A_{c/i}$. Repeating this operation for all the areas, we get to a spatial reassignment of the population (Supplementary Equation 1) into grid cells of masses m_i. This, of course, produces a loss of information leading to a homogenization of some grid cells, which may entirely fall inside the same census area (Supplementary Figure 12) and will have the same population. This is something that does not happen with Twitter data, since it comes directly with coordinates. This means that

$$m_i = \frac{|A_i \cap A_c|}{|A_c|} * m_c. \tag{1}$$

Note that this is not a rescaling of the mass, hence it is only the reassignment of the local population from census areas to regular grid cells of $1 \times 1 km^2$.

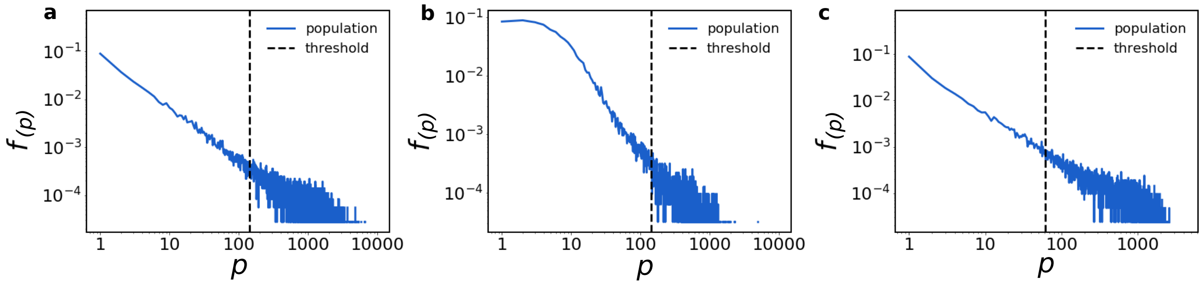

Supplementary Figure 11: **Mass distributions. a** London cells mass distribution. **b** Paris cells mass distribution. **c** Manchester-Liverpool cells mass distribution.

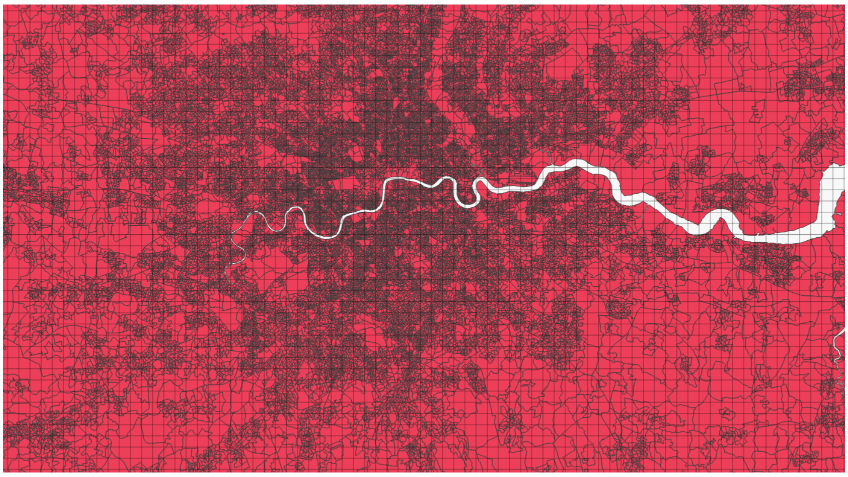

Supplementary Figure 12: **Redistributing census area population into grid cells.** To redistribute the census areas population inside grid cells we used Supplementary Equation 1. The background map with the output areas from the 2011 UK census was obtained from [1] and displayed under a UK Open Government License.

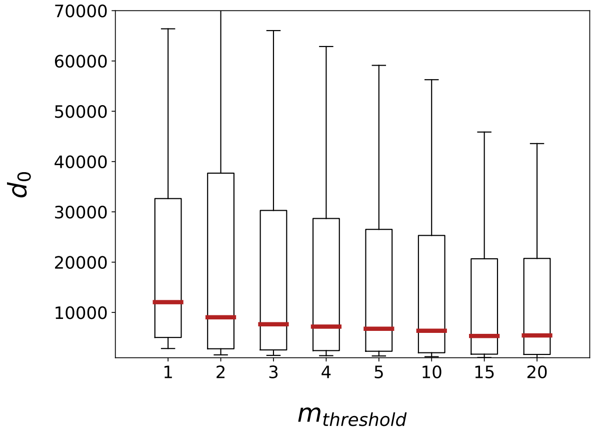

Supplementary Figure 13: **Fitted d_0 distributions boxplot for various thresholds.** Red line to represent the median, box limits to represent the first quartile and the third quartile of the distribution relatively. Whiskers to represent the 15-85 confidence interval. Twitter data.

City	d_0	Population
London (Twitter)	9.40 Km	93,355
Manchester (Twitter)	7.20 Km	33,383
Paris (Twitter)	10.0 Km	28,880
Tokyo (Twitter)	25.0 Km	111,476
Rio de Janeiro (Twitter)	6.30 Km	73,491
Los Angeles (Twitter)	36.0 Km	87,941
London (Census)	7.60 Km	5,652,011
Manchester (Census)	7.20 Km	4,492,149
Paris (Census)	12.0 Km	1,730,059

Supplementary Table 3: List of d_0 fitted parameters and Twitter users per city.

Supplementary Note 3: Fitting the gravity model parameters

We need to fit the parameter d_0 appearing in the deterrence function. In order to do so, we make a list of cells couples i, j and from the general list of cells we extract a third cell x. From this cell we take the empirical flows of commuting people going from x towards the other two cells and divide the two fluxes:

$$\frac{T_{xi}}{T_{xij}} = \frac{k\, m_x\, m_i e^{-d_{xi}/d_0}}{k\, m_x\, m_j e^{-d_{xj}/d_0}} \tag{2}$$

and from this, we get d_0 by saying:

$$d_0 = \frac{d_{xi} - d_{xj}}{-log\left(\frac{T_{xi}\, m_j}{T_{xj}\, m_i}\right)} \tag{3}$$

By doing this, as said above, we set a threshold of minimum mass for the gathering of the cells of the list, in order to avoid strong fluctuations given by underestimated densities. With a statistically robust set of d_0 values, we then proceed to take the median of this parameter, which will be critical in the fits from now on.

As can be seen in Supplementary Figure 13 we find all d_0 in the same order of magnitude in a range between 5 and 10 km, depending on the thresholds by which we subsample the data. We select, therefore, the one maximizing the R_p^2 in the reproduction of the estimated flux Φ_W. Table 3 displays the values of d_0 found for various datasets.

We have adjusted the second parameter k by fitting it to the value that produces the best fluxes in comparison to the empirical data. The values found are listed for the different datasets in Table 4.

City	k
London (Twitter)	$1.61 \times 10^{-5}\ p^{-}1$
Manchester (Twitter)	$5.52 \times 10^{-5}\ p^{-}1$
Paris (Twitter)	$2.58 \times 10^{-5}\ p^{-}1$
Tokyo (Twitter)	$1.03 \times 10^{-5}\ p^{-}1$
Rio de Janeiro (Twitter)	$3.88 \times 10^{-5}\ p^{-}1$
Los Angeles (Twitter)	$9.30 \times 10^{-6}\ p^{-}1$
London (Census)	$2.60 \times 10^{-6}\ p^{-}1$
Manchester (Census)	$7.00 \times 10^{-6}\ p^{-}1$
Paris (Census)	$1.78 \times 10^{-6}\ p^{-}1$

Supplementary Table 4: List of k fitted parameters per city.

Supplementary Note 4: Analytical approaches

In order to facilitate the calculations, we are going to switch to the continuum space. The position of the generic "differential cell" i is at $\vec{\mathbf{r}} = (x, y)$, while the "cell" j will be at $\vec{\mathbf{r}}' = (x', y')$. The mass is then defined in each of this points $m(\vec{\mathbf{r}})$, and the vector field definition in $\vec{\mathbf{r}}$ with the gravity model becomes

$$\vec{\mathbf{W}}(\vec{\mathbf{r}}) = k \int m(\vec{\mathbf{r}}')\, e^{-||\vec{\mathbf{r}}'-\vec{\mathbf{r}}||/d_0}\, \frac{\vec{\mathbf{r}}' - \vec{\mathbf{r}}}{||\vec{\mathbf{r}}' - \vec{\mathbf{r}}||}\, dV', \tag{4}$$

where $||.||$ stands for the modulus of the vector inside. By definition, the flux through a certain closed perimeter C of the vector field $\vec{\mathbf{W}}$ is defined as the path integral over C:

$$\Phi_W(C) = \int_C \vec{\mathbf{W}} \cdot d\vec{\mathbf{n}}, \tag{5}$$

where in each point $\vec{\mathbf{W}} \cdot d\vec{\mathbf{n}}$ is the dot product between the field $\vec{\mathbf{W}}$ and the normal to C. The Gauss (Divergence) Theorem states that for "well behaved" fields this flux must be equal to the volume integral over the space enclosed by C

$$\Phi_W(C) = \int \nabla \cdot \vec{\mathbf{W}}\, dV, \tag{6}$$

where dV is the element of volume and $\nabla \cdot \vec{\mathbf{W}}$ is the field divergence. Note that we are in 2D, so the volume is an area.

Given the definition of the field in Supplementary Equation (4), the divergence of the field produced in a point in space (x, y) by a cell centered at (x_i, y_i) is:

$$\nabla \cdot \vec{\mathbf{W}}_i = k\, m_i \left(\frac{1}{\sqrt{(x - x_i)^2 + (y - y_i)^2}} - \frac{1}{d_0} \right) e^{-\sqrt{(x-x_i)^2+(y-y_i)^2}/d_0}. \tag{7}$$

We are going to consider first a contour that will be a circle around a central point where the cell i is located. Given the symmetries, it is more convenient to express the divergence in polar coordinates:

$$\nabla \cdot \vec{\mathbf{W}}_i = k\, m_i \left(\frac{1}{r} - \frac{1}{d_0} \right) e^{-r/d_0}. \tag{8}$$

Using Gauss theorem, the total flux is then given by the volume integral:

$$\Phi_W = k\, m_i \int_0^{2\pi} \int_0^{R} \left(\frac{1}{r} - \frac{1}{d_0} \right) e^{-r/d_0}\, r\, dr\, d\theta = 2\pi k\, m_i\, R\, e^{-R/d_0}, \tag{9}$$

where R is the radius of the circle C. It is important to bear in mind this expression because it will be a baseline for future solutions.

The same result can be calculated with the surface integral. For this, it is important to take into account that the element of line on the perimeter of the circle can be expressed as $d\ell = R\, d\theta$ where R is the circle radius and $d\theta$ is the element of angle. This implies that

$$\Phi_W = \int_C \vec{\mathbf{W}}\, d\vec{\mathbf{n}} = k\, m_i \int_0^{2\pi} R\, d\theta\, e^{-R/d_0} = 2\pi k\, m_i\, R\, e^{-R/d_0}, \tag{10}$$

which is the same expression as before.

Unfortunately, things are not so simple when the cell i is not in the center of the circle. Let us assume a situation as that shown in Supplementary Figure 14. According to the divergence theorem, the flux is still

$$\Phi_W = k\, m_i \int_0^{2\pi} \int_0^R \left(\frac{1}{d} - \frac{1}{d_0} \right) e^{-d/d_0}\, r\, dr\, d\theta, \tag{11}$$

where d is the distance between $\vec{\mathbf{r}}_0$ and each element of volume within the surface. In polar coordinates, it can be expressed as

$$d = \sqrt{r^2 + r_0^2 - 2\, r_0\, r\, cos(\theta)}, \tag{12}$$

where the position of the cell is (r_0, α_0), the volume element is at (r, α) and, as it can be seen in Supplementary Figure 14, the angle $\theta = \alpha - \alpha_0$.

This integral is not solvable due to a oscillating term $cos(\theta)$ in the square root defining d and that goes in the exponential argument, this brings us to a point of accumulation of singularities in the complex field when integrating. To advance further, we solve it numerically both in the divergence volume integral and in the surface flux integral, which gives us the fluxes shown in Supplementary Figure 15. As can be seen, the curves calculated with the volume and the surface integral are the same as would be expected from the Gauss theorem. Furthermore, the curves for different $\vec{\mathbf{r}}_0$ look similar. There is a negative part when R/d_0 is smaller than the position of the cell r_0. This is because at those radii the cell is outside C and the flux is in-going (negative). Still once the curves cross the x-axis, the behavior seems scalable. The question that we will address then is if there exists a scaling which will collapse all these curves. This is important because it would imply that we could calculate the flux of any population distribution over a circle by simply integrating the flux created by every unit of mass, for which we could have an analytical expression. We know that the case $\vec{\mathbf{r}}_0 = 0$ admits a simple solution, which is a part of this

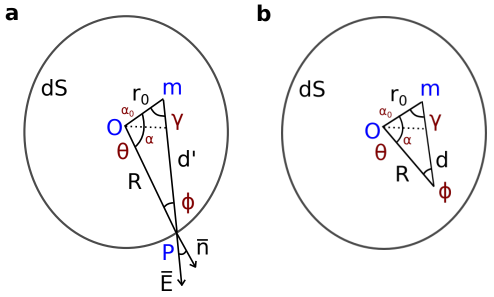

Supplementary Figure 14: **Parameterization scheme.** **a** A perimeter C is drawn as a circle with a population cell i located at m, with distance $\vec{\mathbf{r}}_0$ from the center. To calculate the surface flux, it is necessary to take into account the distance d' to each position on the surface and the angle ϕ with the normal. **b** Same parameterization with the distance from the field generating mass and the observer mass d, to take into account when integrating on the whole volume.

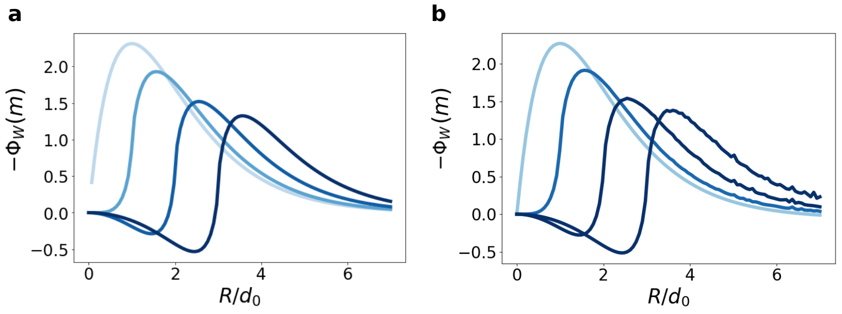

Supplementary Figure 15: **Gauss Theorem** Flux integrals computed taking into account a pointed mass at distance $r_0 = n\, d_0$ with $n = 1, 2, 3, 4$, respectively. $d_0 = 1$ for sake of simplicity. On the x-axis the circle radius of integration R, on the y axis the flux amount. **a** Surface integral. **b** Divergence volume integral.

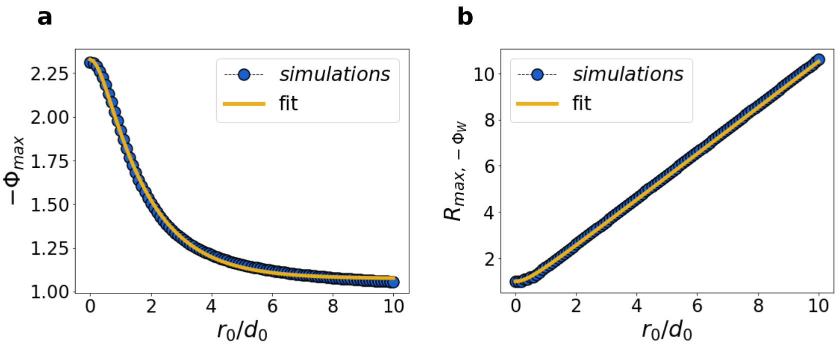

Supplementary Figure 16: **Analytical fits of the maxima.** **a** Maximum of the flux curves Φ_{max} as a function of r_0 and with $d_0 = 1$ for sake of simplicity. We used the function of r_0 derived from the Taylor Expansion (Supplementary Equation 18) to find the corresponding curve to properly fit the behavior of Φ_{max} directly to r_0. We obtained a trend given by $\Phi_{max}(r_0/d_0) = c + a\, e^{-b\, f(r_0/d_0)}$ with $a = 2.41$, $b = 0.66$, $c = 1.07$ and with very small errors. **b** The positions of the maxima of flux R_{max}/d_0 as function of the position of the cell r_0/d_0. The function obtained from the Taylor expansion is over-imposed in solid traits.

family of functions. We study next the behavior of the maximum of the curves for every mass position r_0 and the position of the maximum on the x axis, namely, the radius of integration at which the flux results to be maximum and try to fit the two trends in order to transform every curve to the "universal" one.

To try to find a shape for the curve of Supplementary Figure 16b, the easiest path is to do a Taylor expansion of the flux close to $r_0 = 0$ and to study which is the functional form that comes out of it. As first observations, please note that the curve of R_{max}/d_0 in the Supplementary Figure 16b shows a linear trend for large values of $r_0/d_0 \gg 1$, while the trend is more curved around $r_0 = 0$. To gain further insights, we have to return to the equation that defines the flux, Supplementary Equation (11) and search for the maximum in R. This means to derive the integral respect the variable R, evaluating the result in R_{max}, equating it to zero and then solve the equation for R_{max}. Starting from Eqs. (11) and (12), we perform a Taylor expansion on r_0/d_0 up to first order around $r_0/d_0 = 0$. This yields the expression

$$\Phi_W \approx \frac{k\, m_i}{d_0} \int_0^{2\pi} d\theta \int_0^R r\, dr \left[-1 \pm \frac{d_0}{r} + \frac{d_0\, r_0\, cos(\theta)}{r^2}\right] \left(1 \mp \frac{r_0\, cos(\theta)}{d_0}\right) e^{\pm r/d_0}, \qquad (13)$$

where the first term within the brackets [.] comes from the expansion of d_0/d and the second (plus the exponential) from the expansion of $exp(-d/d_0)$. The angular component can be easily integrated to

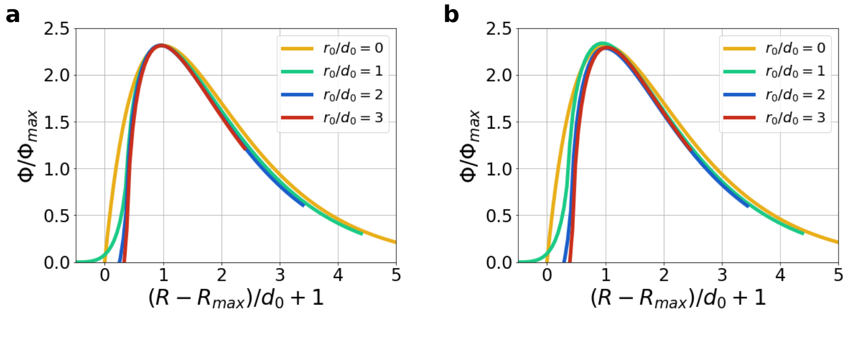

Supplementary Figure 17: **Flux-curves collapse.** Collapse of the curves of the fluxes in Supplementary Figure 15 dividing Φ_W by the corresponding Φ_{max} and changing the x-axis to the empirical value of $(R - R_{max})/d_0$. **a** Empirical collapse. In this case Φ_{max} and R_{max} are found numerically, without fits or approximations, for each r_0 (as in Supplementary Figure 16). **b** Analytical collapse. In this case Φ_{max} and R_{max} are found analytically.

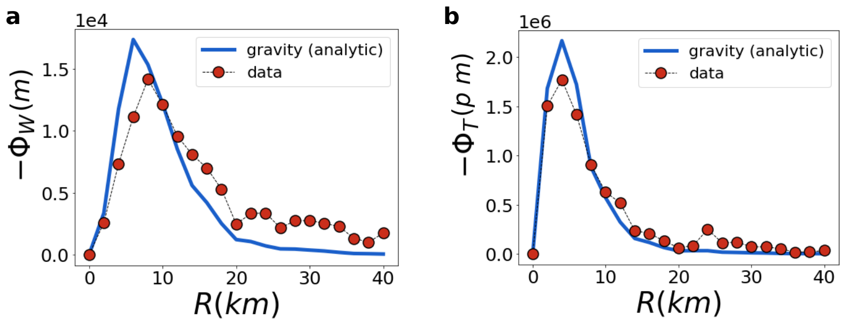

Supplementary Figure 18: **Semi-analytical fluxes in London.** **a** Semi-analytical approach for flux of W. $R_p^2 = 0.66$. **b** Semi-analytical approach for flux of T. $R_p^2 = 0.98$.

obtain

$$\Phi_W \approx \frac{k\, m_i\, \pi}{d_0} \int_0^R r\, dr \left(-2 \pm \frac{2\, d_0}{r} \mp \frac{r_0^2}{r^2}\right) e^{\pm r/d_0}. \tag{14}$$

This is a complicated integral because it diverges (and not in an easy way) at $r = 0$, which is part of the integral domain. Additionally, we have to apply now the Fundamental Theorem of the integral calculus while taking the derivative respect to R. The theorem validity is not ensured in the presence of divergences in the domain of integration. Even so and with all the prevention, if we take the derivative and equate it to zero to find R_{max} the equation becomes

$$\frac{d\Phi_W}{dR}(R_{max}) = \frac{k\, m_i\, \pi}{d_0} R_{max} \left(-2 \pm \frac{2\, d_0}{R_{max}} \mp \frac{r_0^2}{R_{max}^2}\right) e^{\pm R_{max}/d_0} = 0. \tag{15}$$

This equation has several solutions but we are interested in one of them, namely,

$$R_{max} = \frac{d_0}{2} \left(1 + \sqrt{1 + 2\,(r_0/d_0)^2}\right). \tag{16}$$

Note that this function is linear in the limit $(r_0/d_0) \gg 1$, which is an interesting trait given that we know that this is the proper trend in the asymptotic limit. However, the slope in this equation would be $\sqrt{2}/2$ instead of 1 as occurs in the simulations. This failure is normal given that the approach employed is valid (if at all) only in the range of $(r_0/d_0) \ll 1$.

Inspired by these results, we are going to propose an ansatz for R_{max} in the full range of values of r_0/d_0. The functional form must be almost equal to that of Supplementary Equation (16) to maintain the good behavior in the limit $r_0/d_0 \to 0$, but the final slope must be 1. Motivated by the fact that in high order expansions the parameters of the expression of R_{max} change, we are going to try with the following arbitrary function:

$$R_{max} = \frac{d_0}{2} \left(1 + \sqrt{1 + 4\,(r_0/d_0)^2}\right). \tag{17}$$

The only difference is a factor 4 instead of 2 multiplying r_0/d_0 under the square root. Curiously, it turns out that the fit is quite good in all the domain of values of r_0/d_0 if one plots this function over the numerical values of R_{max} (see Supplementary Figure 16b). We will use it as an approximation for R_{max} in the coming plots.

The height of the maxima of the flux curves of Supplementary Figure 15 is shown in Supplementary Figure 16a as a function of the radius R/d_0. This scaling is important to obtain a general formula for the flux regardless of the distribution of the population. The curve for Φ_{max} seems to have a behavior related to that of R_{max}. For small values of r_0/d_0, there is a very short plateau followed by a exponential decay. The interval where functional trend changes coincides with that of R_{max}. This leads to hypothesize that Φ_{max} is a function of R_{max}, which means that we can write it as a function of the expression (17) of r_0. Given that this expression has a linear growth and Φ_max an exponential decay, we are going to try with

$$\Phi_{max}(r_0/d_0) = c + a\, e^{-b\, R_{max}} = c + a\, e^{-b\, d_0\, \left(1+\sqrt{1+4\,(r_0/d_0)^2}\right)/2}, \tag{18}$$

where the best fit has occurred for the values of $a = 2.41$, $b = 0.662$ and $c = 1.07$.

The first question to check is that, as we hypothesized, the flux curves for different positions of the cell i, r_0/d_0, can be collapsed if we rescale the y-axis to Φ/Φ_{max} and the x-axis to $(R - R_{max})/d_0$ (see Supplementary Figure 17a). In fact, these values approximated for Φ_{max} and R_{max} have been used to do a second collapse of the curves in Supplementary Figure 15. The results of taking Φ_W/Φ_{max} and $(R - R_{max})/d_0 + 1$ are shown in Supplementary Figure 17b. As can be seen, the curves collapse well even if they have slightly different shapes. Other way to check the quality of these results is to analyze the flux as a function of the radius as predicted by the approximation and that measured from the empirical data. This is done in Supplementary Figure 18 both for the flux Φ_W (a) and for the total flow out of the circle Φ_T (b). The method to obtain these plots is by using the individual functions of the flux for the locations of each cell r_0, its corresponding population m_i and sum all of them to obtain the flux produced by the heterogeneous population of the city.

Supplementary Note 5: The gravity model with a power-law deterrence function

The gravity model with an exponential decay in the deterrence function provides the best results in our analysis when compared with the empirical data. However, the most common functional shape for the deterrence function in the literature is a power-law decaying function. In this case, the gravity law becomes:

$$T_{ij} = k\,\frac{m_i\,m_j}{d_{ij}^{\gamma}}, \tag{19}$$

where γ is the exponent, which must be $\gamma \geq 1$ to ensure that the total flow is integrable. The case $\gamma = 1$ deserves special attention because it corresponds to the gravitational or electric fields in a two dimensional space. In this notation for the vector field, we have:

$$\vec{\mathbf{T}}_i = k\,m_i \sum_j \frac{m_j}{d_{ij}^{\gamma}}\,\vec{\mathbf{u}}_{ij}, \tag{20}$$

which implies that the field is

$$\vec{\mathbf{W}}_i = k \sum_j \frac{m_j}{d_{ij}^{\gamma}}\,\vec{\mathbf{u}}_{ij}. \tag{21}$$

In the continuum, this becomes:

$$\vec{\mathbf{W}}_i = k \int \frac{m(\vec{\mathbf{r}}')}{d_{ij}^{\gamma}}\,\vec{\mathbf{u}}_{ij}\,dV. \tag{22}$$

Numerical results

Again, as we did before, we try to reproduce the flux at different radii from the city center by measuring the field produced by every cell population in the urban zone given by the power law deterrence function. The results are shown in Supplementary Figures 19 and 20. As can be seen, this type of deterrence function yields worse results than the exponential one. Specially the flux at short scales (low R) is underestimated.

In Supplementary Figure 19a, we are considering the case of $\gamma = 1$ using as mass the information obtained from the Twitter data. Note that we have applied the numerical technique described in the Methods Section of the manuscript to calculate the fluxes. This explains why the flux decays for large values of R, which are distances far away from the city center, where the population of commuters is scarce and almost no vectors contribute to Φ_W.

Analytical results

Let us consider first the situation in which the population m_i is located at the center of the circle C with radius R. In this case, the flux over the circle can be directly calculated as the surface integral:

$$\Phi_W = \int_C \vec{\mathbf{W}}\,d\vec{\mathbf{n}} = k\,m_i \int_0^{2\,pi} R\,d\theta\,R^{-\gamma} = 2\,\pi\,k\,m_i\,R^{1-\gamma}. \tag{23}$$

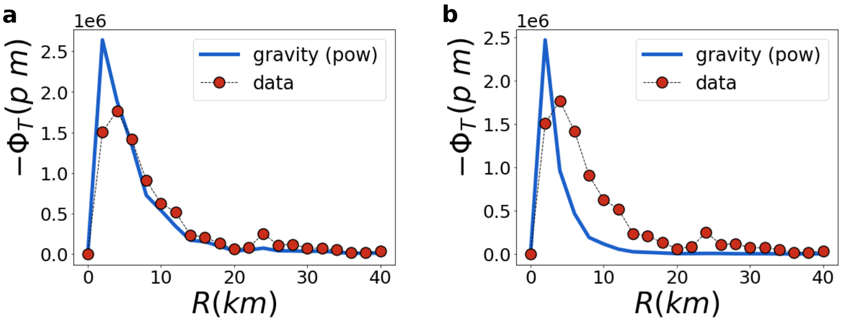

Supplementary Figure 19: **Flux analysis in London.** Power law case Gravity Model predicted fluxes of the vector field T. **a** $\gamma = 1.$ $k = 0.04$ $R_p^2 = 0.90$ **b** $\gamma = 2.$ $k = 60$ $R_p^2 = 0.60$

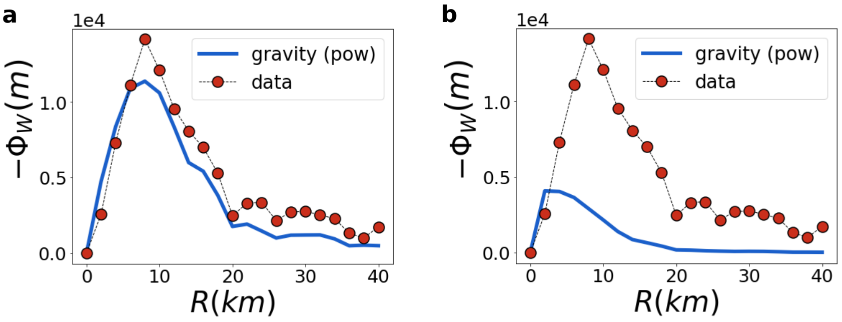

Supplementary Figure 20: **Power-law fluxes analysis in London.** Power law case Gravity Model predicted fluxes of the vector field W. **a** $\gamma = 1.$ $k = 0.04$ $R_p^2 = 0.92$ **b** $\gamma = 2.$ $k = 60$ $R_p^2 = 0.40$

This solution is valid for all the values of R and γ except if $\gamma > 1$ and R tends to zero where there is a divergence. In the special case $\gamma = 1$, the flux Φ_W depends only on the total mass enclosed by the circle. For the analytical calculations, we are using the continuous approach and assuming a value of the field in every point. This explains the difference respect to the numerical integration shown in Supplementary Figure 19a.

As before, we can also use the Gauss Theorem to calculate the flux performing the volume integral of the divergence of the field. However, this is relatively complicated for this case because the divergence of $\vec{\mathbf{W}}$ strongly depends on γ. If $\gamma < 1$, the divergence is

$$\nabla \cdot \vec{\mathbf{W}}(\vec{\mathbf{r}}) = k\, m_i\, \frac{(1-\gamma)}{d^{\gamma+1}}. \tag{24}$$

If $\gamma = 1$, then

$$\nabla \cdot \vec{\mathbf{W}}(\vec{\mathbf{r}}) = 2\,\pi\, k\, m_i\, \delta(\vec{\mathbf{r}}). \tag{25}$$

We are not sure how to define it for larger values of γ. Calculating the volume integral, the divergence of $\vec{\mathbf{W}}$ yields:

$$\Phi_W = k\, m_i \int_0^{2\,\pi} d\theta \int_0^R \frac{(1-\gamma)}{r^{\gamma+1}}\, r\, dr = 2\,\pi\, k\, m_i\, R^{1-\gamma}, \tag{26}$$

for $\gamma < 1$. Similarly, for $\gamma = 1$ the result is

$$\Phi_W = 2\,\pi\, k\, m_i \int_V dV\, \delta(\vec{\mathbf{r}}) = 2\,\pi\, k\, m_i, \tag{27}$$

fulfilling Maxwell's law.

For a cell located at a distance r_0 of the center at an angle α_0, the calculation is not simple. In polar coordinates and following the notation of Supplementary Figure 14, we can write that

$$d' = \sqrt{r_0^2 + R^2 - 2\, R\, r_0\, cos(\theta)}, \tag{28}$$

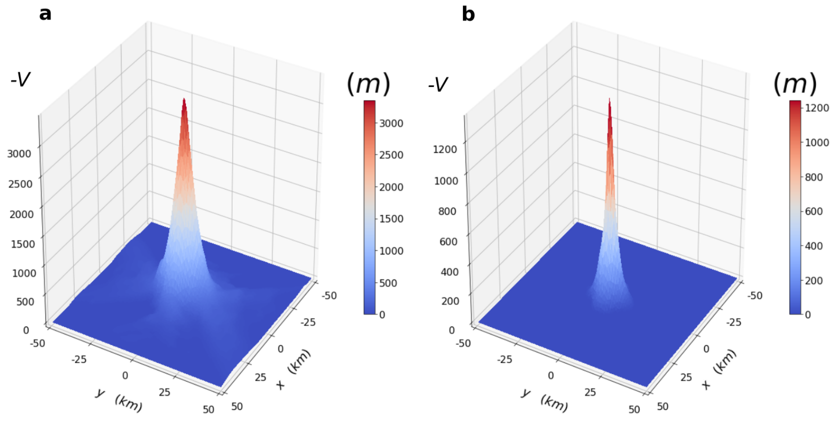

Supplementary Figure 21: **Power-law potentials analysis.** Predicted city potential with power law deterrence function Gravity model. **a** $\gamma = 1$. **b** $\gamma = 2$.

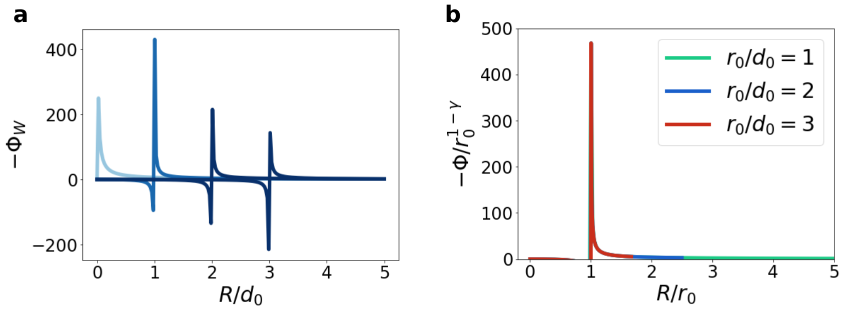

Supplementary Figure 22: **Power-law flux curves collapse.** Flux curves for different mass position r_0 with power law deterrence function exponent. **a** Simulated fluxes for different mass position r_0 with power law deterrence function exponent $\gamma = 2$ **b** Analytical collapse.

where R is again the radius of the circle. The surface integral can then be written as

$$\Phi_W = m_i\, k \int_0^{2\,\pi} R\, d\theta \frac{cos(\phi)}{[r_0^2 + R^2 - 2\, R\, r_0\, cos(\theta)]^{\gamma/2}}, \tag{29}$$

where the angle ϕ is the one formed between the normal of the circle and the vector between the position of the population and the element of surface for which the integral is being calculated. It can be express as a combination of θ, R and d:

$$cos(\phi) = \frac{R - r_0\, cos(\theta)}{d'}. \tag{30}$$

Replacing $cos(\phi)$ under the integral, it becomes

$$\Phi_W = m_i\, k \int_0^{2\,\pi} R\, d\theta \frac{R - r_0\, cos(\theta)}{[r_0^2 + R^2 - 2\, R\, r_0\, cos(\theta)]^{(\gamma+1)/2}}. \tag{31}$$

Dividing by r_0 in the numerator and denominator, we obtain

$$\Phi_W = m_i\, k\, r_0^{1-\gamma} \int_0^{2\,\pi} u\, d\theta \frac{u - cos(\theta)}{[1 + u^2 - 2\, u\, cos(\theta)]^{(\gamma+1)/2}}, \tag{32}$$

where $u = R/r_0$ and it is the only free parameter under the integral. This means that the result of the integral is a function only of u and, therefore, the flux can be written as scaling functions of the type:

$$\Phi_W = r_0^{1-\gamma}\, f_\gamma(R/r_0), \tag{33}$$

which is an exact solution in contrasts to what happened in the exponential case. An example of the curves of Φ_w for $\gamma = 2$ is displayed at Supplementary Figure 22. The curves are collapsed in Supplementary Figure 22b.

The integral of Supplementary Equation (32) can be solved analytically as long $r_0 \neq R$, yielding

$$\Phi_W = m_i\, k\, r_0^{1-\gamma}\, \pi\, u \left\{ \frac{u}{(-1+u)^{(1+\gamma)}}\, {}_2F_1\left(\frac{1}{2}, \frac{1+\gamma}{2}, 1, \frac{-4\,u}{(-1+u)^2}\right) + \frac{u}{(1+u)^{(1+\gamma)}}\, {}_2F_1\left(\frac{1}{2}, \frac{1+\gamma}{2}, 1, \frac{4\,u}{(1+u)^2}\right) \right.$$
$$- \frac{1}{(1+u)^2\,(-1+u)^{(1+\gamma)}} \left[-3\,(-1+u)^2\, {}_2F_1\left(\frac{-1}{2}, \frac{1+\gamma}{2}, 2, \frac{-4\,u}{(-1+u)^2}\right) \right.$$
$$\left. +(1+u)^2\, {}_2F_1\left(\frac{1}{2}, \frac{1+\gamma}{2}, 1, \frac{-4\,u}{(-1+u)^2}\right) + 2\,(1+2\,(-1+\gamma)\,u+u^2)\, {}_2F_1\left(\frac{1}{2}, \frac{1+\gamma}{2}, 2, \frac{-4\,u}{(-1+u)^2}\right) \right]$$
$$+ \frac{1}{(-1+u)^2\,(1+u)^{(1+\gamma)}} \left[-3\,(1+u)^2\, {}_2F_1\left(\frac{-1}{2}, \frac{1+\gamma}{2}, 2, \frac{4\,u}{(1+u)^2}\right) \right.$$
$$\left.\left. +(u-1)^2\, {}_2F_1\left(\frac{1}{2}, \frac{1+\gamma}{2}, 1, \frac{4\,u}{(1+u)^2}\right) + 2\,(1-2\,(-1+\gamma)\,u+u^2)\, {}_2F_1\left(\frac{1}{2}, \frac{1+\gamma}{2}, 2, \frac{4\,u}{(1+u)^2}\right) \right] \right\},$$

where ${}_2F_1(.)$ is Gauss hypergeometric function. The function between $\{.\}$ depends only on u and γ as expected, but its particular form is not very informative.

As in the exponential case, the potential for the city can be obtained from the field. The potential for a force created in a cell i by a set of cells j can be written as

$$V_i = k \sum_j m_j \frac{d_{i,j}^{1-\gamma}}{(1-\gamma)}. \tag{34}$$

Computing this quantity from the population distribution from our data, we get a potential peaked in the city center. As can be seen in Supplementary Figure 21 for $\gamma = 2$ the potential is too weak and it does not compare so well with the empirical one as the curve produced by the exponential deterrence function. On the other hand, for $\gamma = 1$ the potential gets closer to the one found from the data and the exponential case, still the fluxes show that this functional form is not the optimal one to reproduce the data.

Supplementary Note 6: Gravity vs Radiation: model comparison

We show the resulting vector field of commuting people in the city of London, as computed through the first version of the Radiation Model. As shown, this vector field is not comparable with the empirical one observed from the data (see Supplementary Figure 23). The curl is indeed almost zero everywhere and this would allow us to define a potential in this case too. Nonetheless the analysis of the simulated flux of people passing through the different circles at different radiuses is much lower than the empirical one and gives worse correlations, as shown in Supplementary Figure 24.

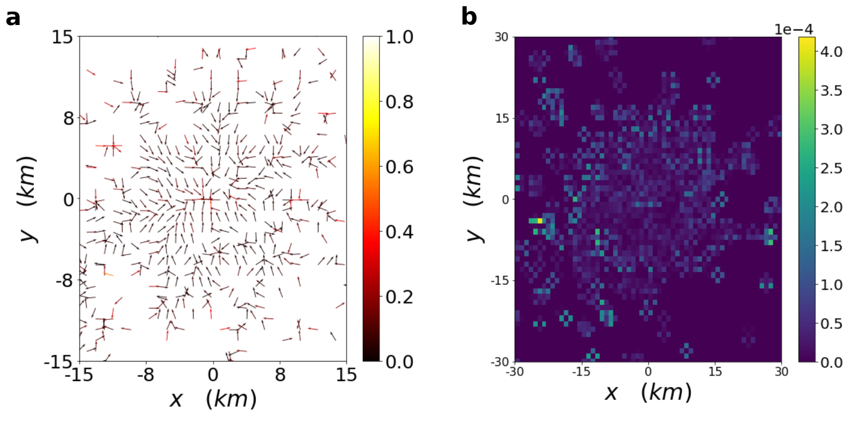

Supplementary Figure 23: **Radiation Model predicted vector field in London.** **a** The vector field resulted by the Radiation Model. **b** The curl modulus of the relative vector field W computed for the Radiation Model vectors in m^{-1}.

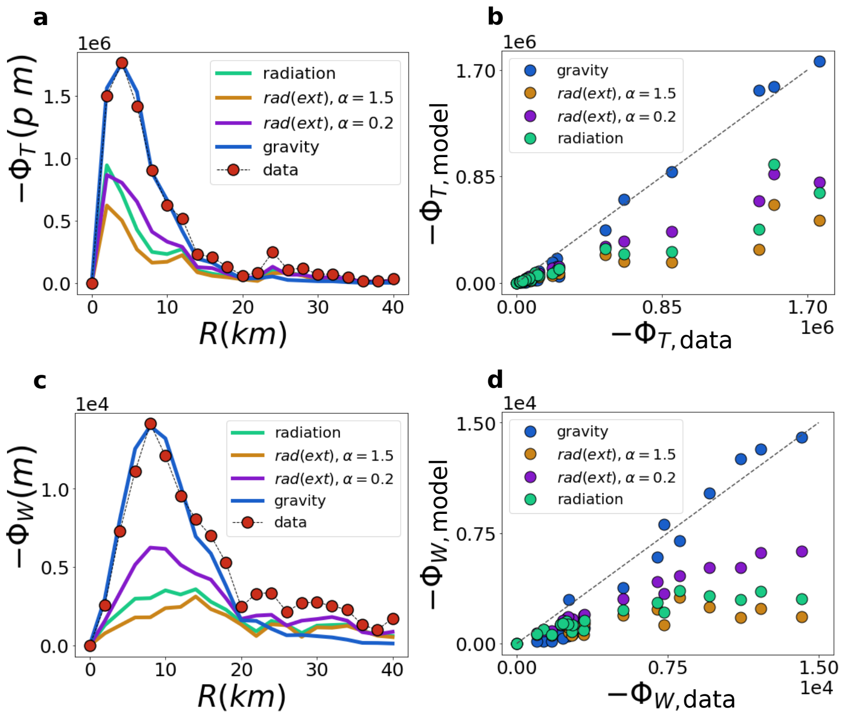

Supplementary Figure 24: **Model comparison in London.** **a,b** Fluxes of the vector field T. **c,d** Fluxes of the vector field W.

We compare the gravity with the exponentially decaying deterrence function with the Radiation Model for further cities and with the nonlinear version of the Radiation Model proposed by Yang et al. [2]. The non-linear attractivity of this last version of the Radiation Model summarizes into the formula:

$$\langle T_{ij} \rangle = \frac{[(m_i + m_j + s_{ij})^{\alpha} - (m_i + s_{ij})^{\alpha}]\,({m_i}^{\alpha} + 1)}{[(m_i + s_{ij})^{\alpha} + 1]\,[(m_i + m_j + s_{ij})^{\alpha} + 1]}, \tag{35}$$

with α as the only parameter of the model. In our results, we used different values of α in the range $\alpha \in [0.2, 1.5]$. For the sake of visualization, we will only show the results with $\alpha = 0.2$ and $\alpha = 1.5$. All the values of α in between of these two values gave flux curves in between of the two shown for each city, with no better accuracy observed in any of the cases.

What we see from the plots of the Radiation Model applied to London, Los Angeles and Tokyo is that, it systematically underestimates the flux in all the cases. The extended version of the Radiation Model works slightly better with $\alpha = 0.2$, but still the model underestimates the fluxes even in this case.

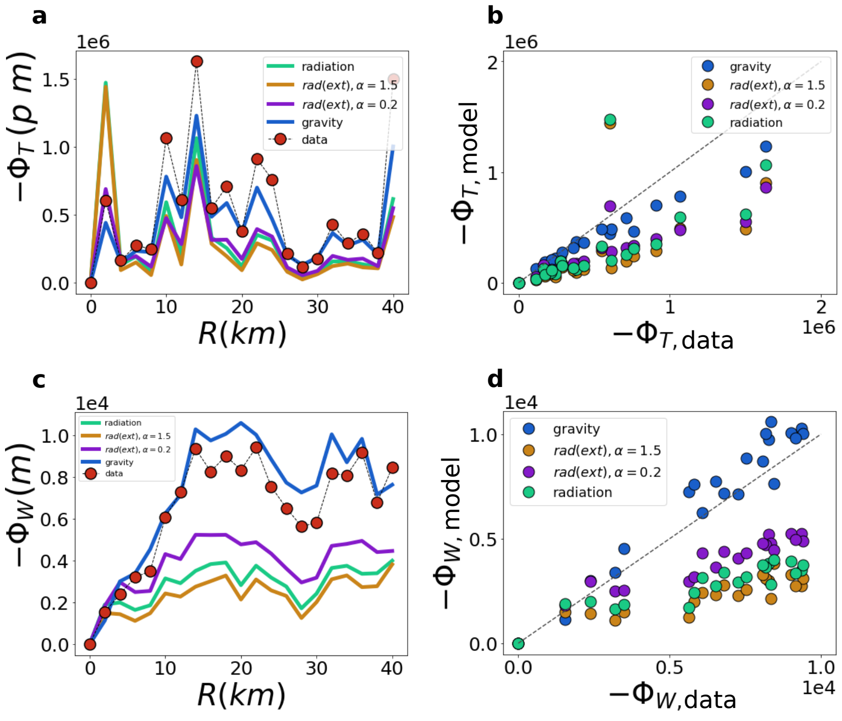

Supplementary Figure 25: **Model comparison in Los Angeles.** **a,b** Fluxes of the vector field T. **c,d** Fluxes of the vector field W.

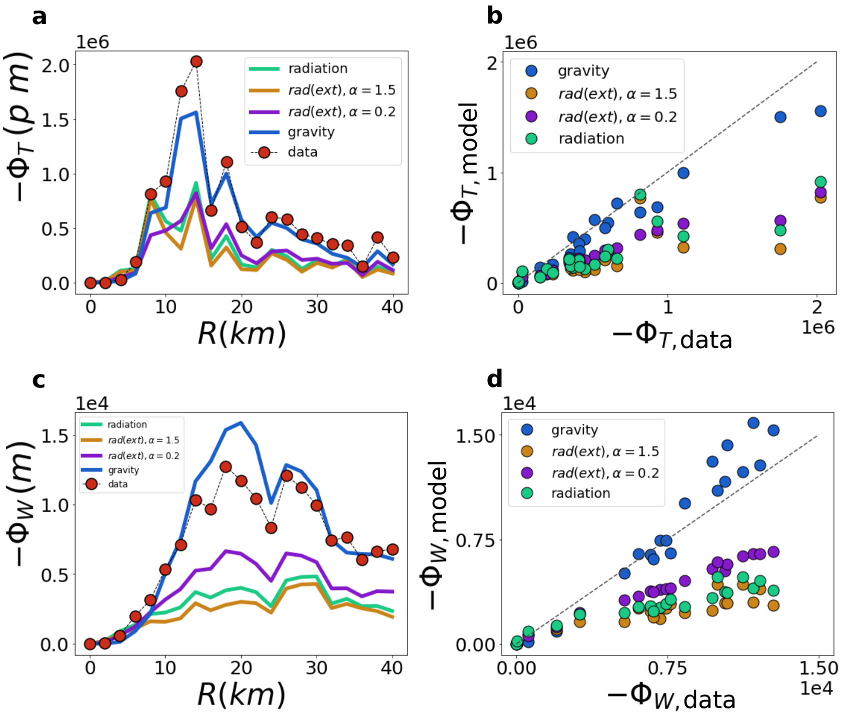

Supplementary Figure 26: **Model comparison in Tokyo.** **a,b** Fluxes of the vector field T. **c,d** Fluxes of the vector field W.

Supplementary Note 7: Further cities with Twitter data

Manchester - Liverpool - Leeds - Sheffield

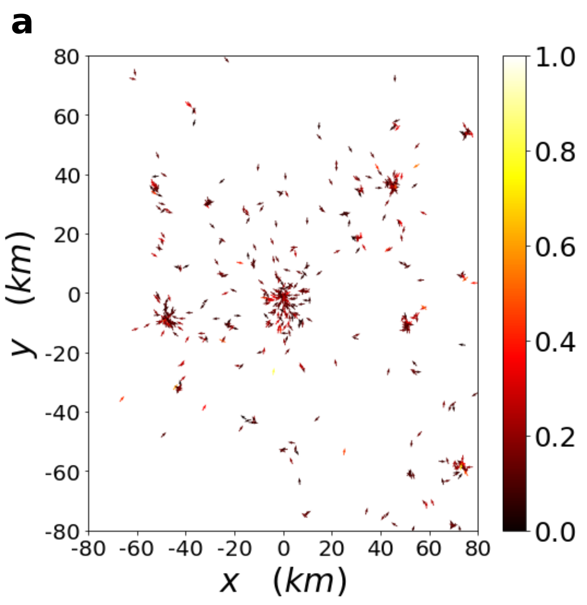

Supplementary Figure 27: **Vector fields comparison in Manchester.** **a** City empirical vector field. **b** City Gravity model predicted vector field.

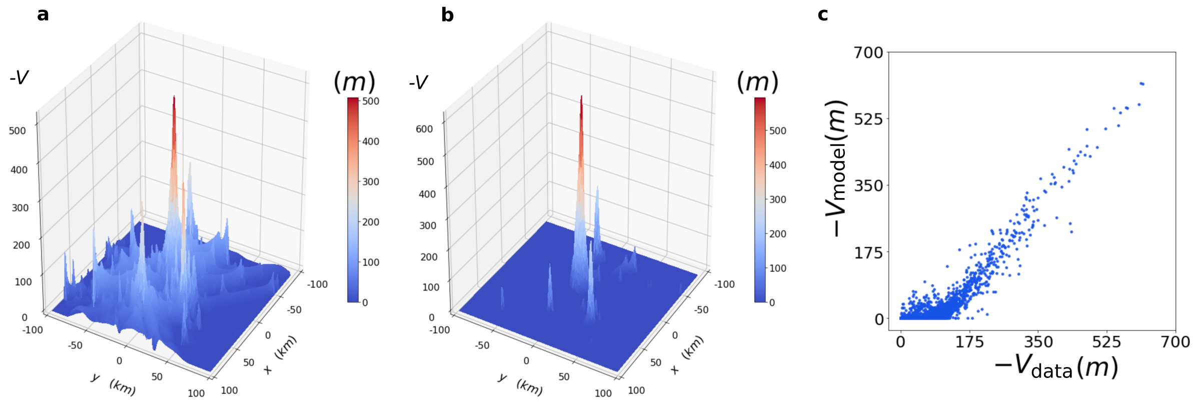

Supplementary Figure 28: **Potentials comparison in Manchester.** **a** City empirical potential. **b** City Gravity model predicted potential. **c** Potentials correlation plot. $R_p^2 = 0.81$.

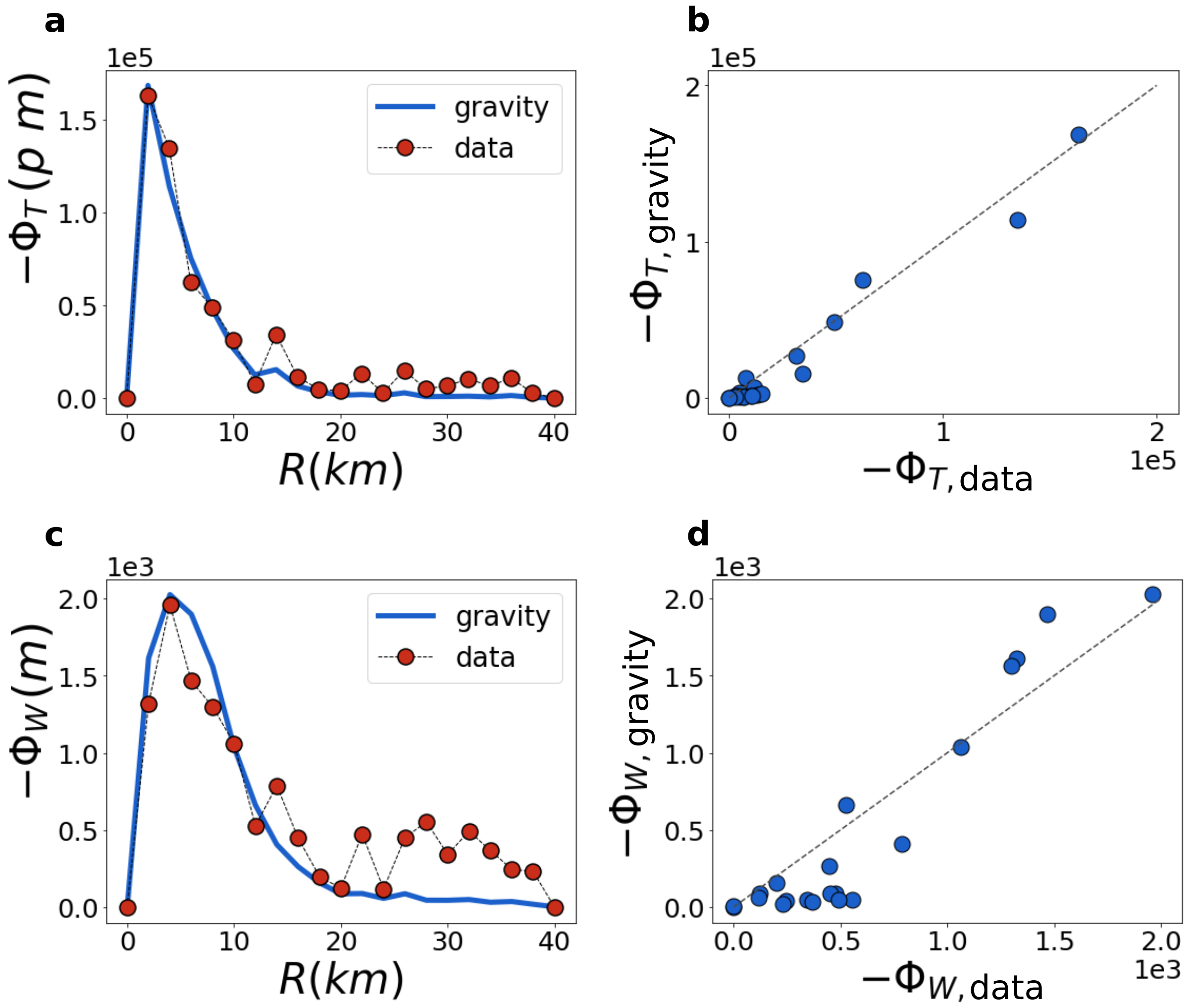

Supplementary Figure 29: **Fluxes comparison in Manchester.** **a,b** Fluxes of the vector field T. $R_p^2 = 0.97$. **c,d** Fluxes of the vector field W. $R_p^2 = 0.89$.

Paris

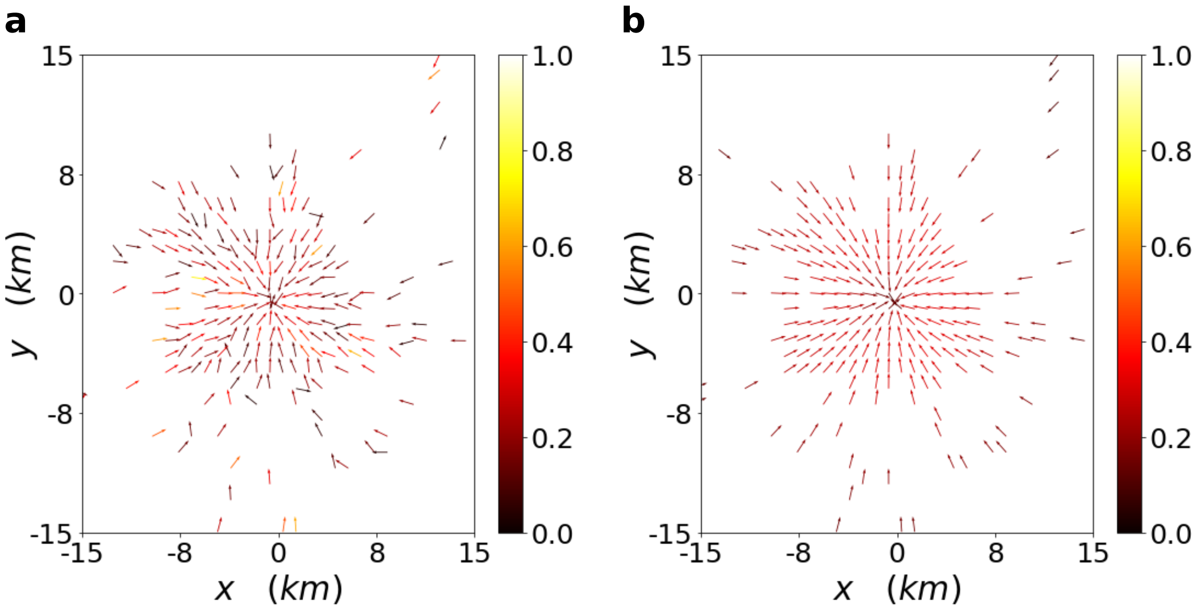

Supplementary Figure 30: **Vector fields comparison in Paris.** **a**City empirical vector field. **b** City Gravity model predicted vector field.

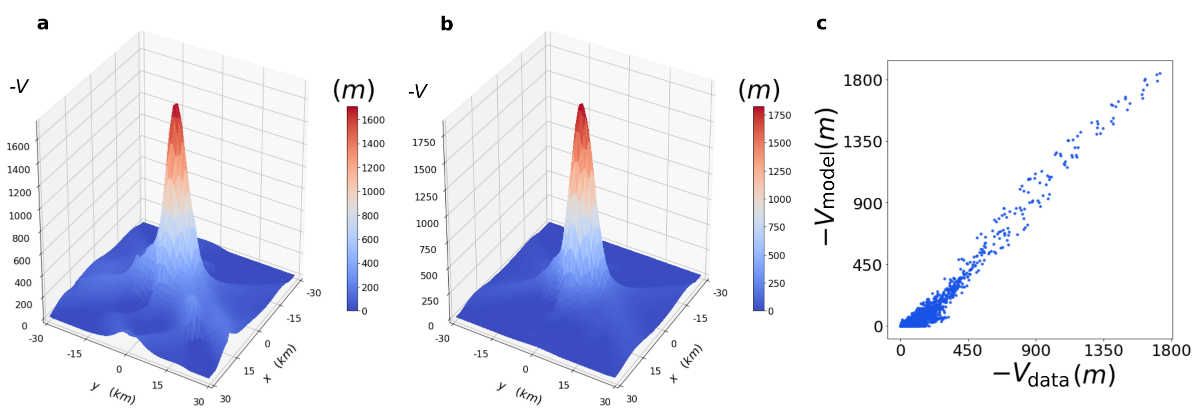

Supplementary Figure 31: **Potentials comparison in Paris.** **a** City empirical potential. **b** City Gravity model predicted potential. **c** Potentials correlation plot. $R_p^2 = 0.96$.

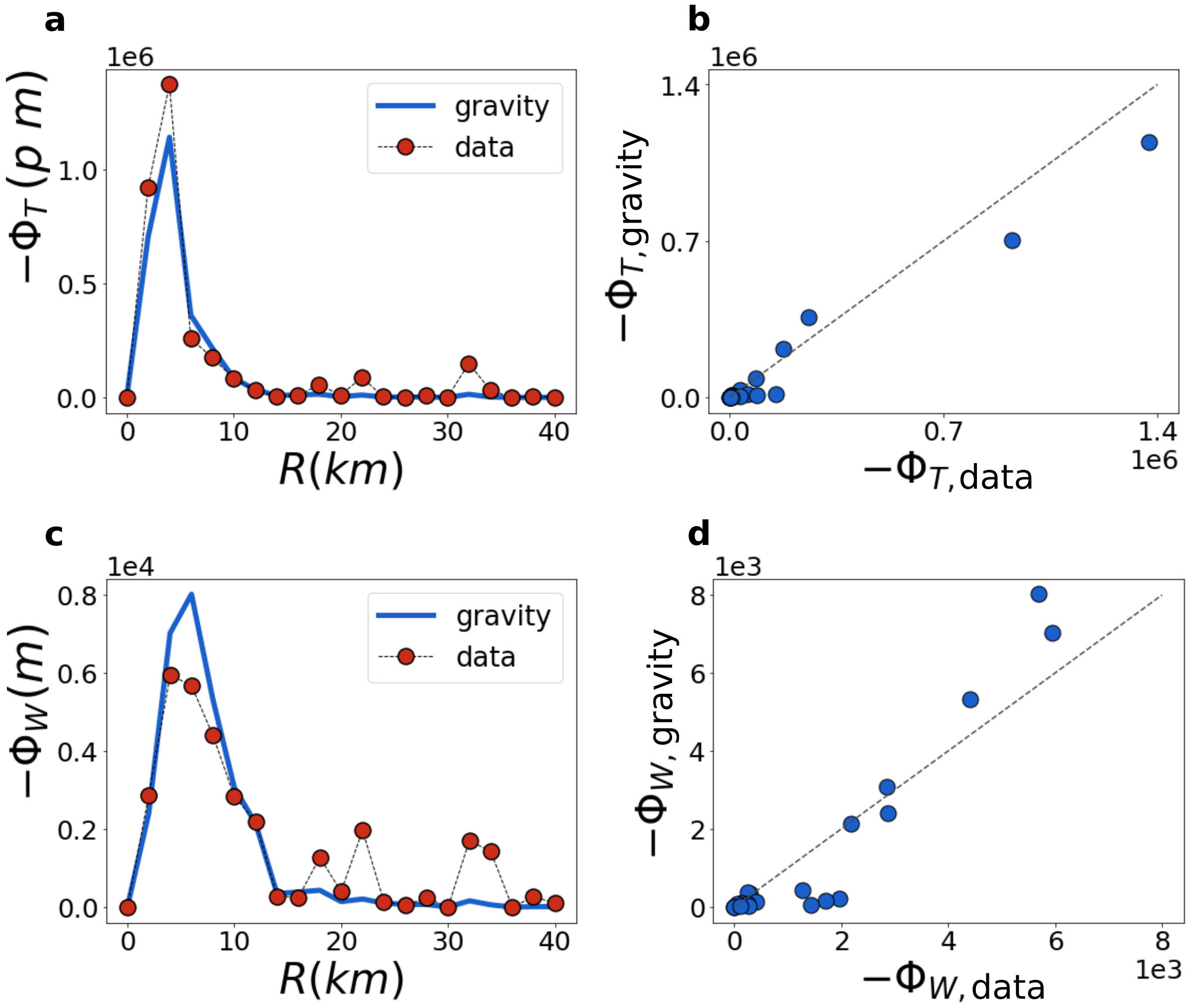

Supplementary Figure 32: **Fluxes comparison in Paris. a,b** Fluxes of the vector field T. $R_p^2 = 0.97$. **c,d** Fluxes of the vector field W. $R_p^2 = 0.90$.

Los Angeles

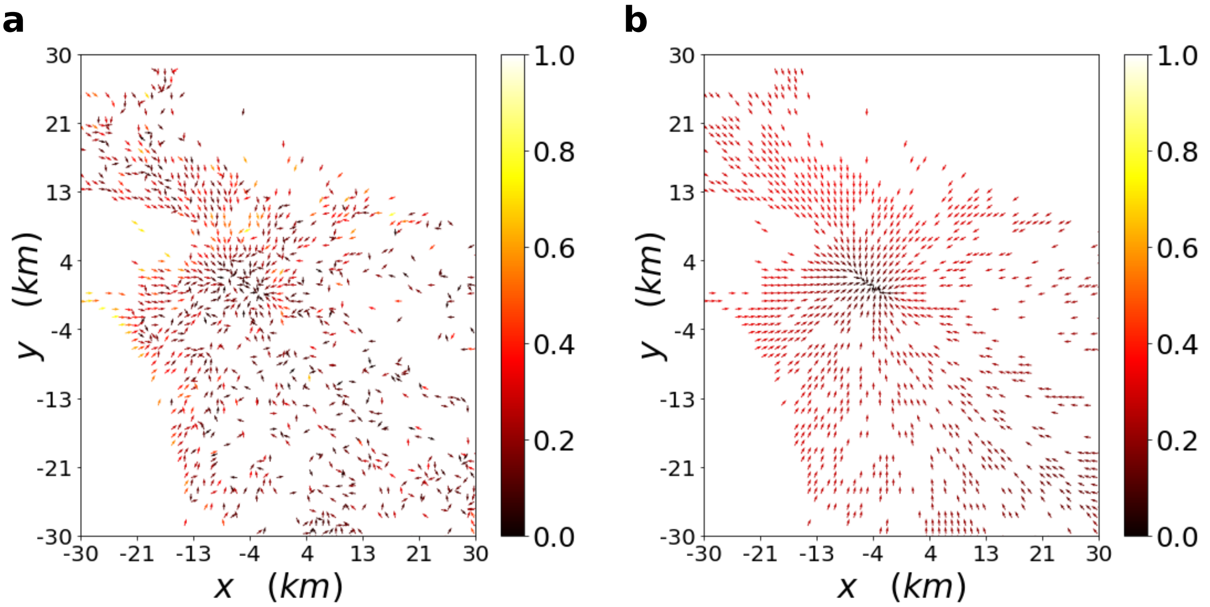

Supplementary Figure 33: **Vector fields comparison in Los Angeles.** **a**City empirical vector field. **b** City Gravity model predicted vector field.

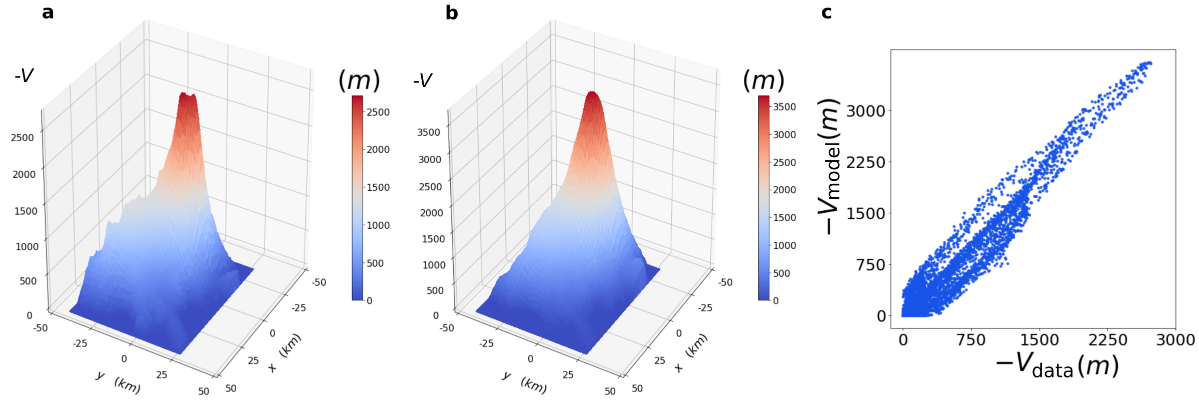

Supplementary Figure 34: **Potentials comparison in Los Angeles.** **a** City empirical potential. **b** City Gravity model predicted potential. **c** Potentials correlation plot. $R_p^2 = 0.93$.

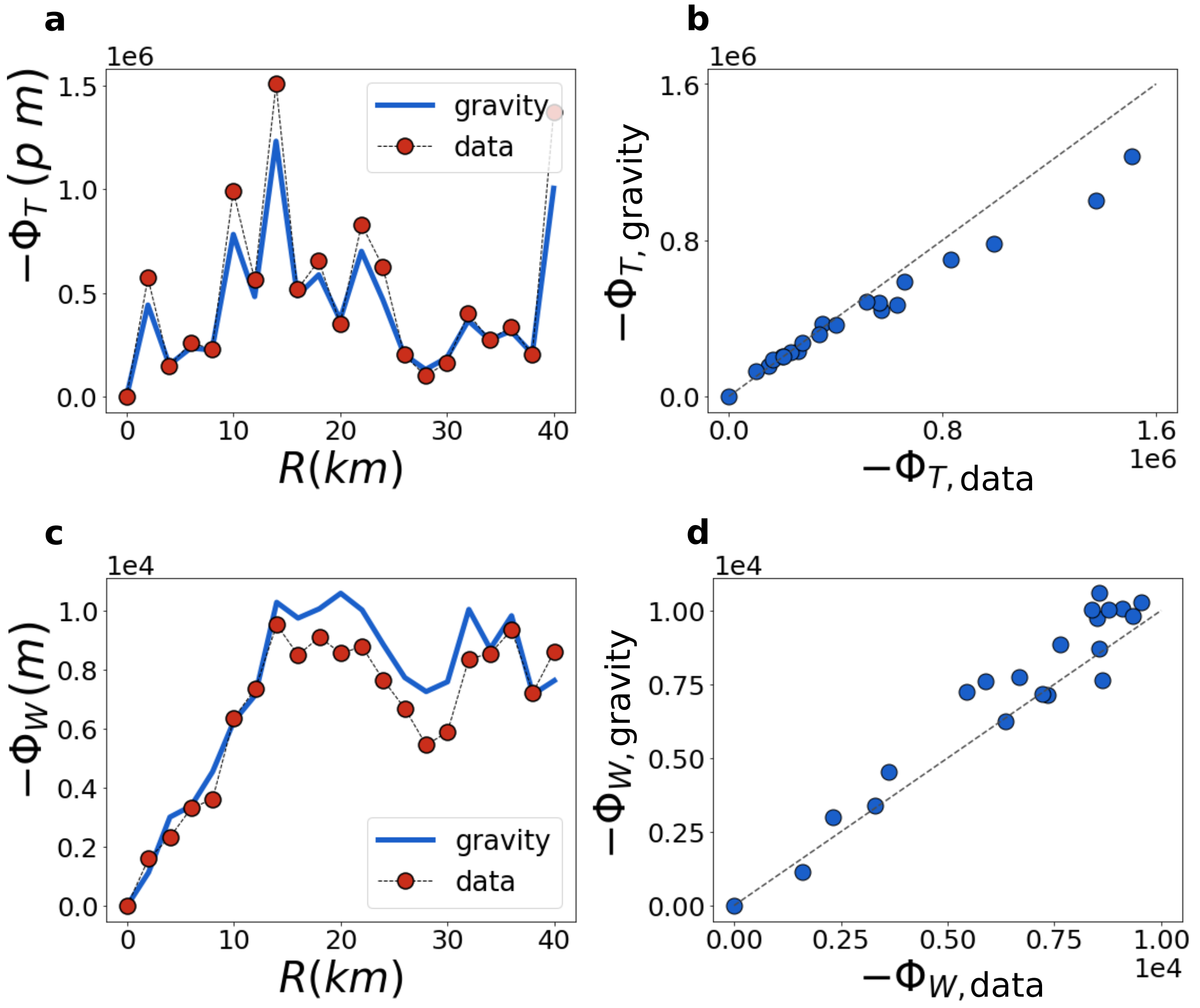

Supplementary Figure 35: **Fluxes comparison in Los Angeles.** **a,b** Fluxes of the vector field T. $R_p^2 = 0.99$. **c,d** Fluxes of the vector field W. $R_p^2 = 0.93$.

Tokyo

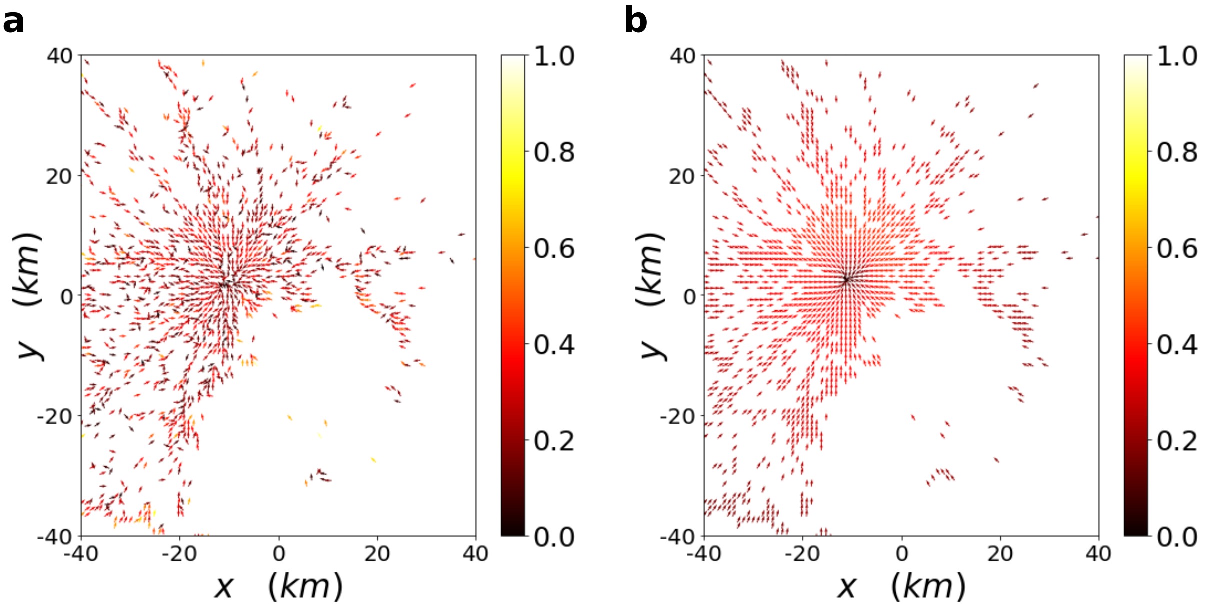

Supplementary Figure 36: **Vector fields comparison in Tokyo.** **a**City empirical vector field. **b** City Gravity model predicted vector field.

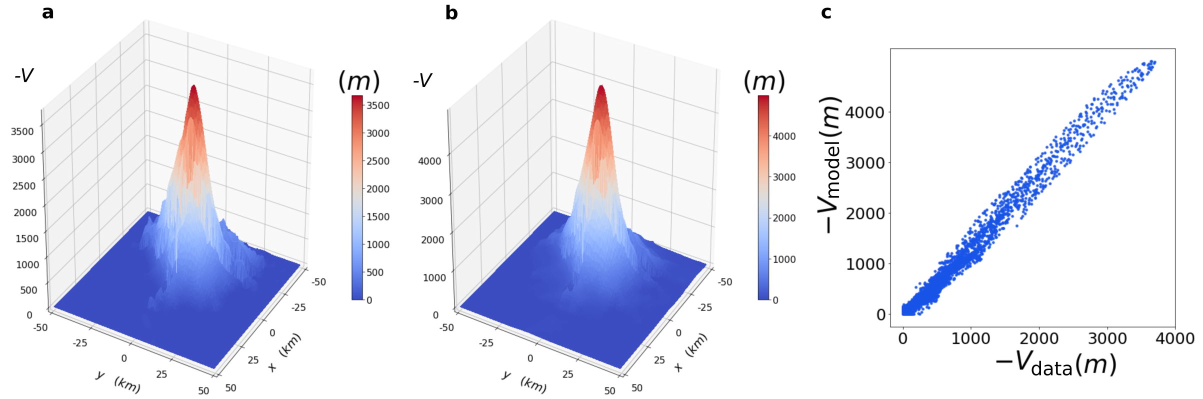

Supplementary Figure 37: **Potentials comparison in Tokyo.** **a** City empirical potential. **b** City Gravity model predicted potential. **c** Potentials correlation plot. $R_p^2 = 0.98$.

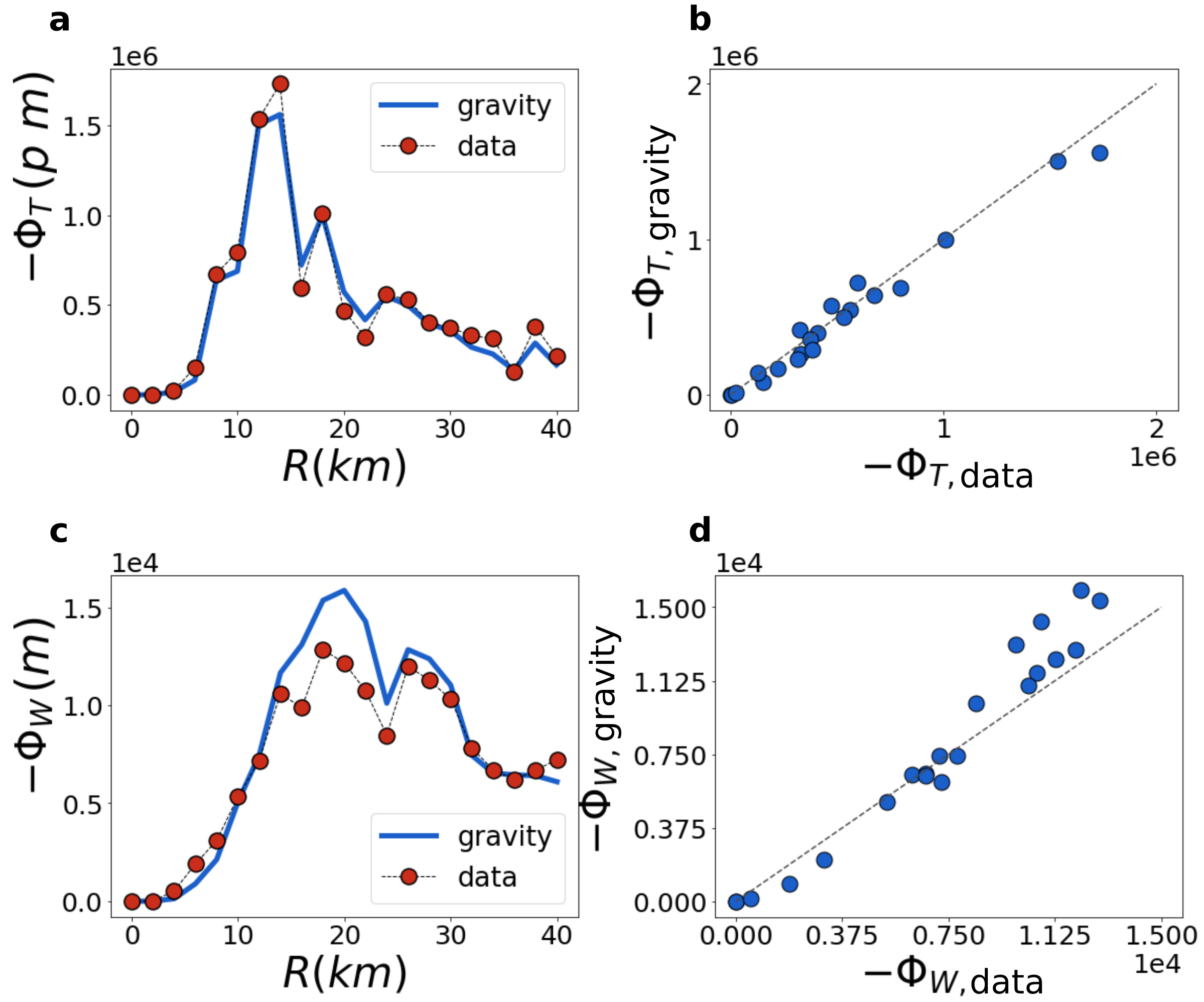

Supplementary Figure 38: **Fluxes comparison in Tokyo. a,b** Fluxes of the vector field T. $R_p^2 = 0.98$. **c,d** Fluxes of the vector field W. $R_p^2 = 0.96$.

Rio de Janeiro

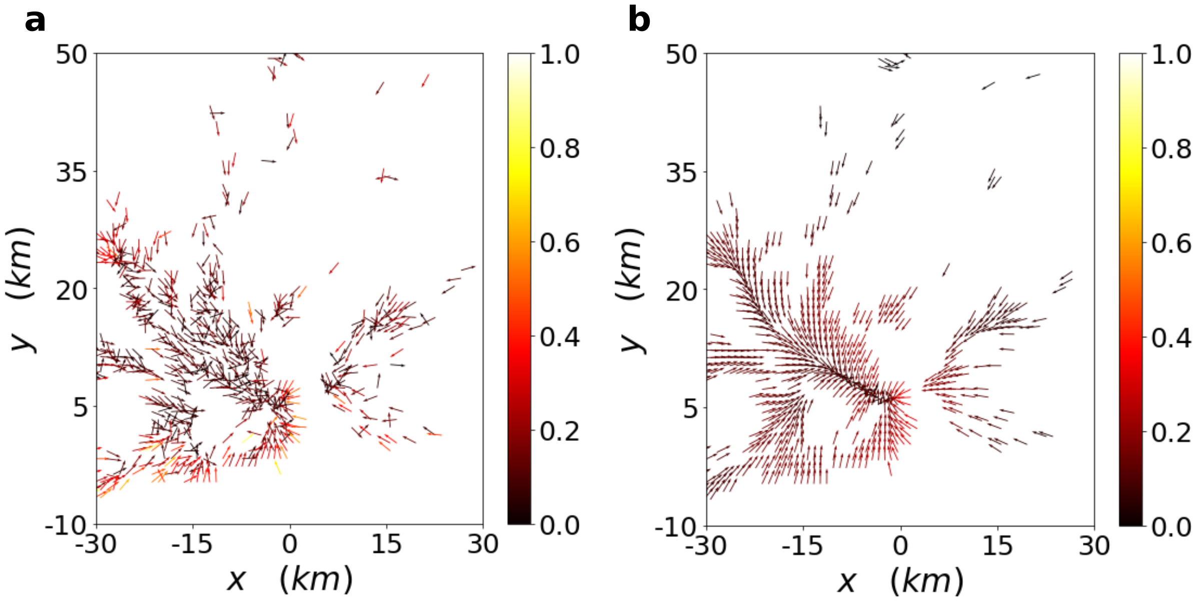

Supplementary Figure 39: **Vector fields comparison in Rio.** **a**City empirical vector field. **b** City Gravity model predicted vector field.

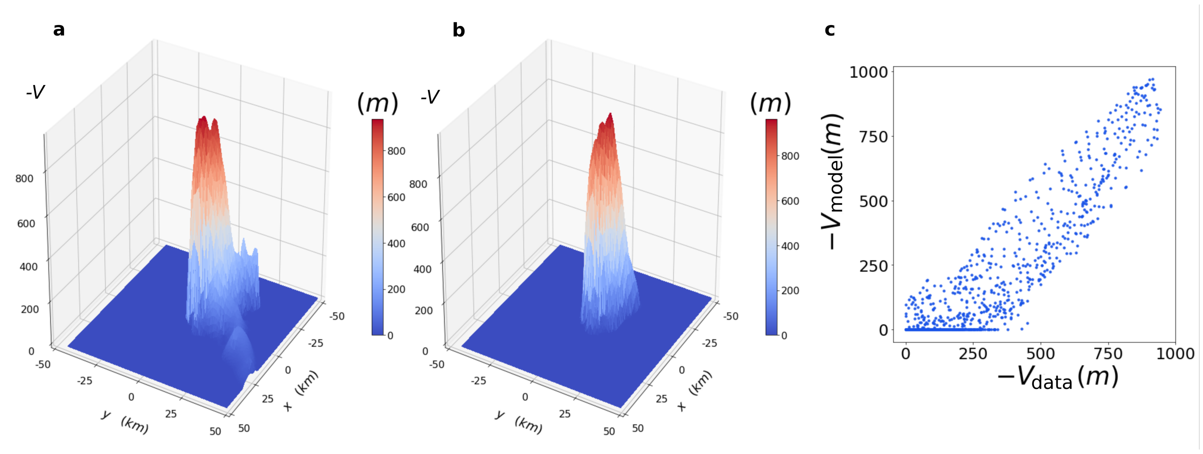

Supplementary Figure 40: **Potentials comparison in Rio.** **a** City empirical potential. **b** City Gravity model predicted potential. **c** Potentials correlation plot. $R_p^2 = 0.87$.

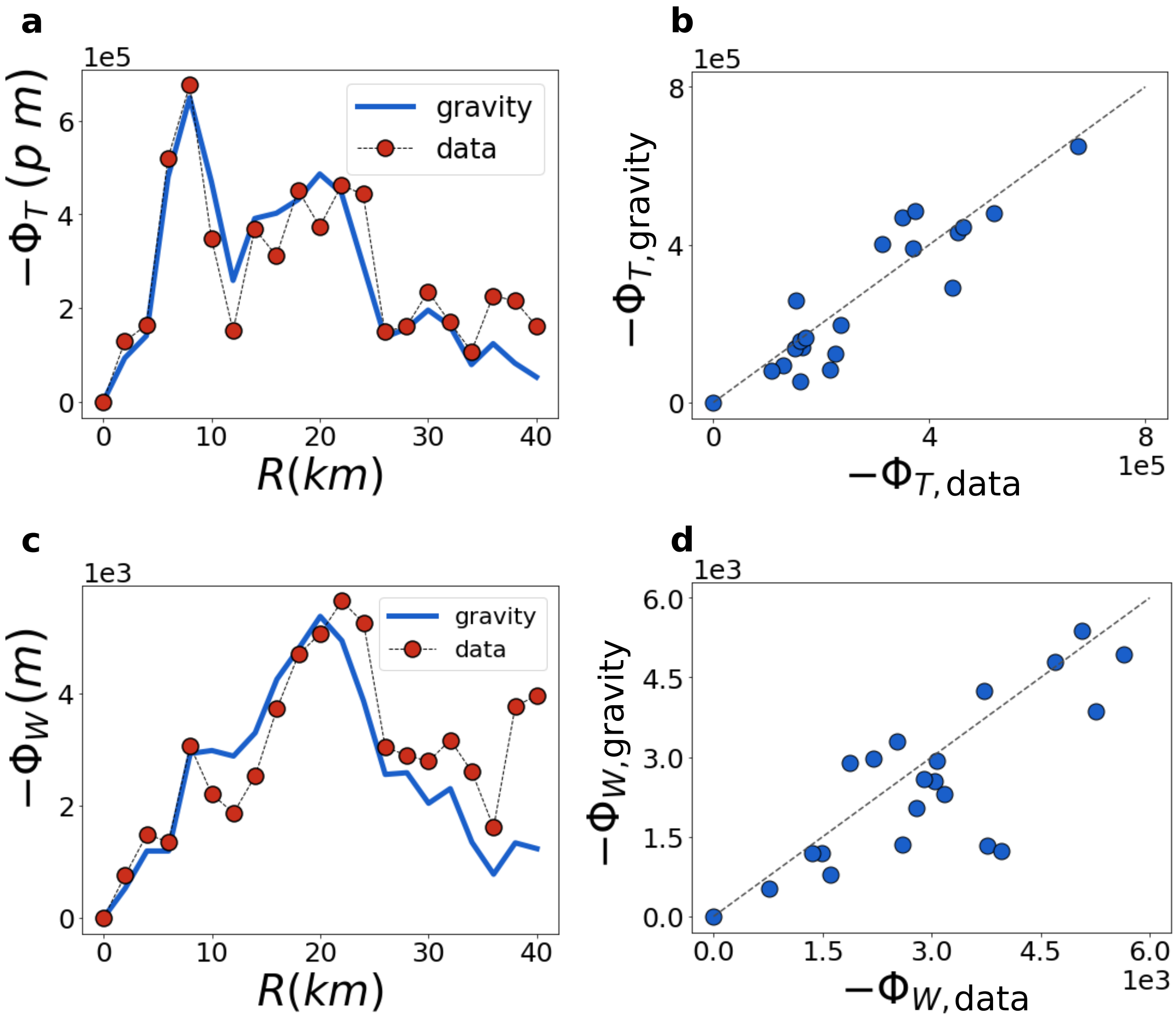

Supplementary Figure 41: **Fluxes comparison in Rio. a,b** Fluxes of the vector field T. $R_p^2 = 0.83$. **c,d** Fluxes of the vector field W. $R_p^2 = 0.63$.

Supplementary Note 8: Further equipotential curves

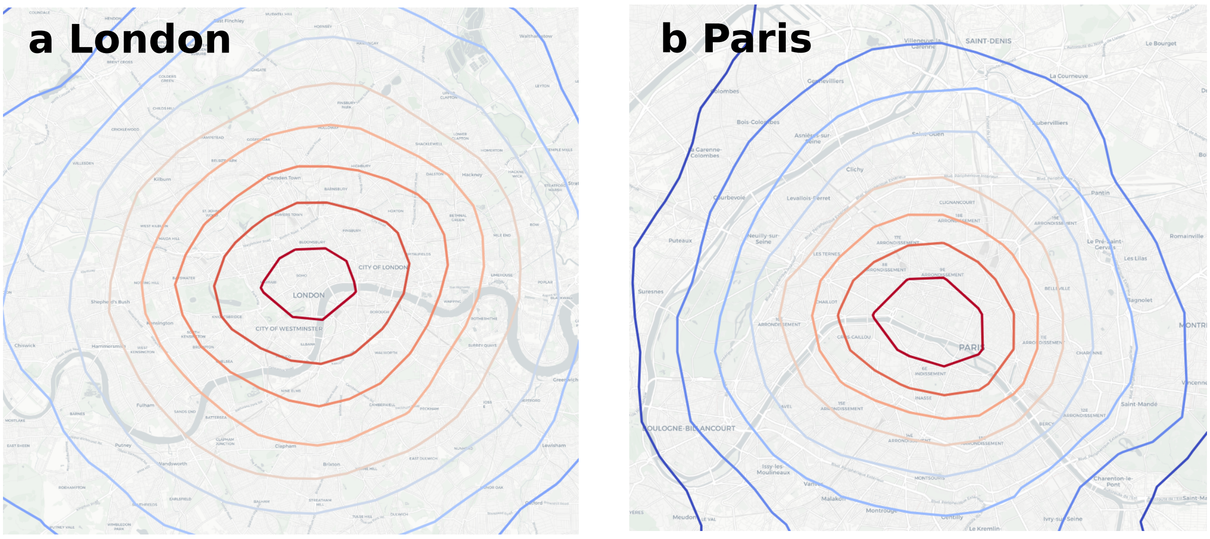

Supplementary Figure 42: **Empirical equipotential lines.** **a** London **b** Paris. The underground map layout is produced using Stamen Toner. Map tiles by Stamen Design, under CC BY 3.0. Data by OpenStreetMap, under ODbL.

Supplementary Note 9: Fluctuations in the empirical curl

In order to explore the fluctuations of the empirical curl of $\vec{\mathbf{W}}$ in London, we show the plot using a logarithmic scale on the y-axis. A parabolic fit has been applied to the distribution with correlation $R_p^2 = 0.75$ to check if it was compatible with a Gaussian. As can be seen in Supplementary Figure 43, the distribution is a composition of randomly distributed noise around a delta function peaked at $\nabla \times \vec{\mathbf{W}} = 0$ and a symmetrical exponential decay in each direction. In fact, the distribution for the curl of the null model (right) has the same shape but it is wider.

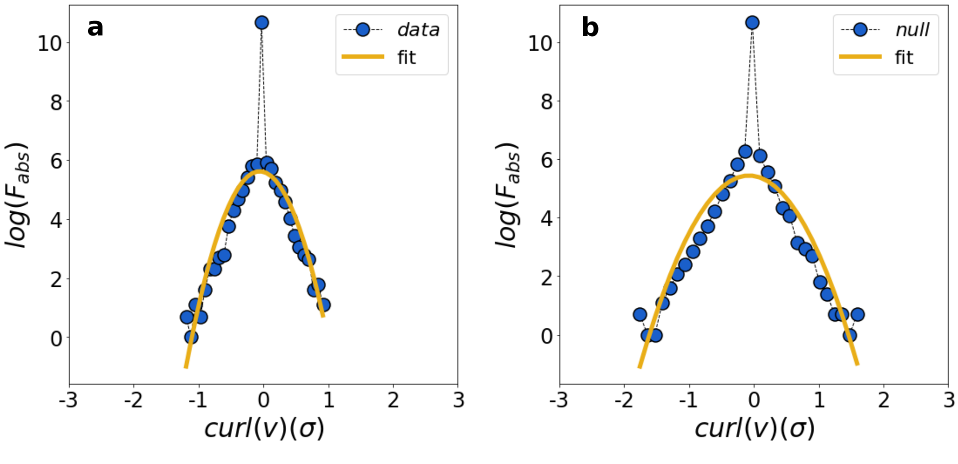

Supplementary Figure 43: **Curl values distribution in London.** Empirical field (left) and the null model (right).

Supplementary Note 10: Trip distance distribution

As a comparison with previous works on this field we show here the empirical trip distance distributions we get in London, Paris and Los Angeles and compare them with those estimated by the gravity model with exponential deterrence function and those estimated by the radiation model.

As can be seen in Fig.44, it is not clear from this metric which model fits best the empirical distribution of trips (except in Los Angeles). As already shown in a previous comprehensive work [3] the gravity model

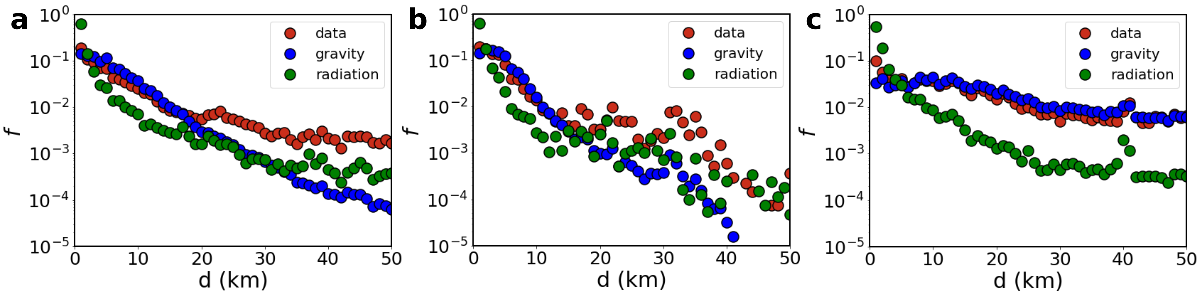

Supplementary Figure 44: **Trip distance distributions a** London. **b** Paris. **c** Los Angeles.

works better at short distances while the radiation model works better at long distances. Moreover, the gravity model parameters are tuned on the mesoscale behavior of the space cells, hence on the locally aggregated behavior of commuters living in a determined neighborhood. This does not happen with the radiation model where the information on each individual can be retrieved in a stochastic process.

Supplementary Note 11: Angle distribution

To prove once more the irrotationality of our vector field we show here the commuting vector field angle distribution for London, Paris and Los Angeles. As one would expect from an irrotational field, the vectors are all directed towards the minimum of the potential, hence the distribution is plain. To show this better we compute the complementary cumulative density functions for each city, which is linear, proving the uniform contribution of each bin in the histogram, as shown in Fig.46.

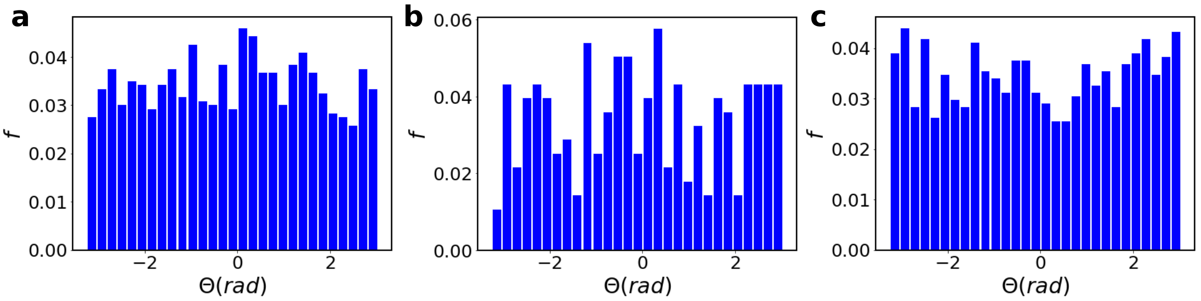

Supplementary Figure 45: **Vector-field angle distributions. a** London, mean value $\overline{\Theta} = 9 \times 10^{-4} rad$, variance $\sigma^2_{\Theta} = 3.12 rad^2$. **b** Paris, mean value $\overline{\Theta} = 8.63 \times 10^{-2} rad$, variance $\sigma^2_{\Theta} = 3.22 rad^2$. **c** Los Angeles, mean value $\overline{\Theta} = 2.76 \times 10^{-2} rad$, variance $\sigma^2_{\Theta} = 3.51 rad^2$.

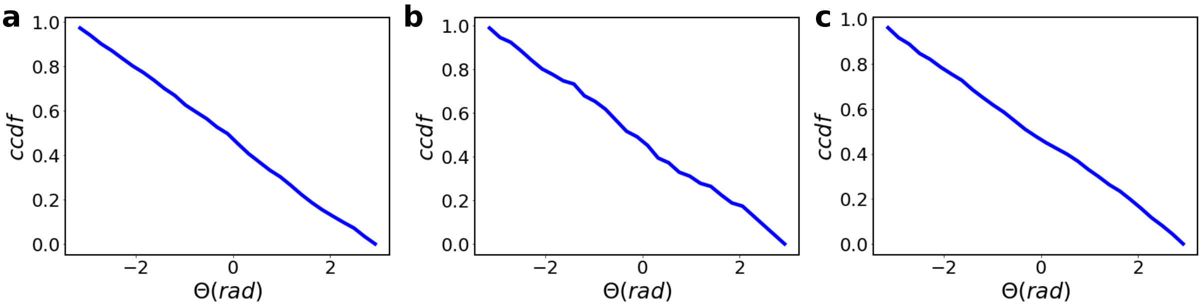

Supplementary Figure 46: **Vector field angles.** Complementary cumulative distribution (ccdf) for **a** London. **b** Paris. **c** Los Angeles.

Angle distribution can be a critical metric for better discerning models performances. As shown in Fig.47 the gravity model is the best at catching the directionality of the aggregated commuting

mobility flows, while the radiation gives sparser results. It is important to note that, given whatever parameterization, e.g. $\Theta \in [-\pi, \pi]$ yields some inconsistencies, which is to say that model vectors resulting in $\Theta_{model} = -3.1 rad$ look like differing a lot from an empirical vector with $Theta_{empirical} = 3.12$, when they differ only for few decimals of a radiant. However, this mainly occurs only in Los Angeles, while on a general trend, it is clear that the gravity model outcompetes the radiation model, as the R_p^2 values show.

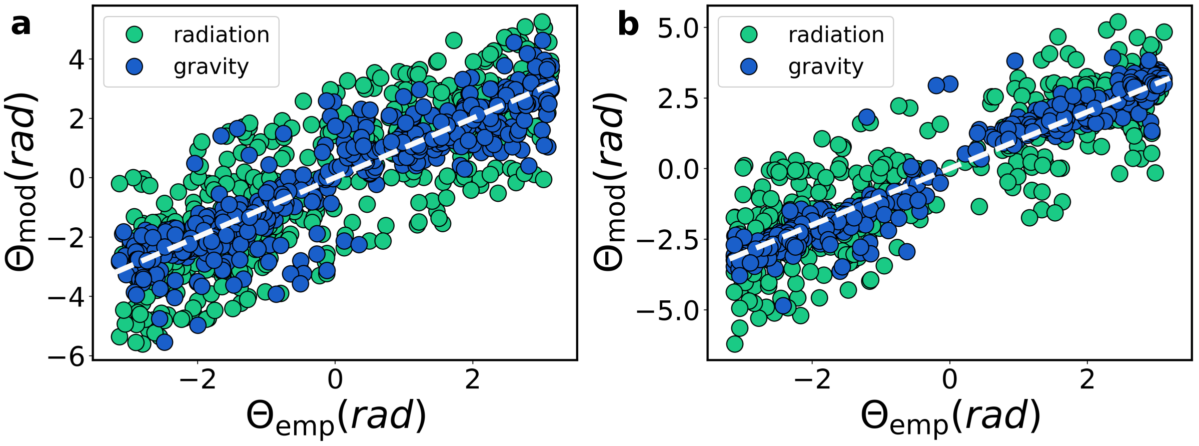

Supplementary Figure 47: **Vector field angle comparison.** **a** Los Angeles, gravity model $R_p^2 = 0.83$, radiation model $R_p^2 = 0.55$. **b** Tokyo, gravity model $R_p^2 = 0.94$, radiation model $R_p^2 = 0.67$.

In Fig.48 we show the angular first module difference among the empirical outflow vectors of each cell. This vectors are those who have to be vectorially summed up to generate the resultant vectors for each cell. This is done in order to avoid angles parameterization issues, i.e. given the parameterization $\Theta \in [-\pi, \pi]$, as said before, we would find a great difference between $\Theta_{model} = -3.1 rad$ and $Theta_{empirical} = 3.12$, when they differ only for few decimals of a radiant. To avoid this, we compute:

$$\Delta = \Sigma_{i \neq j} |\Theta_i - \Theta_j| / n. \tag{36}$$

where n is the total number of permutations once can get from the set of vectors in each cell. Every time we take the difference between each couple of angles as the "shortest path" on the unit circle. This Δ represents the average window of outflows directions one can take from each cell. As shown by Fig.48 the first module difference behaves the same in the three cities, as explained by the almost uniform color. Hence we can say we do not observe particular features by looking at this quantity.

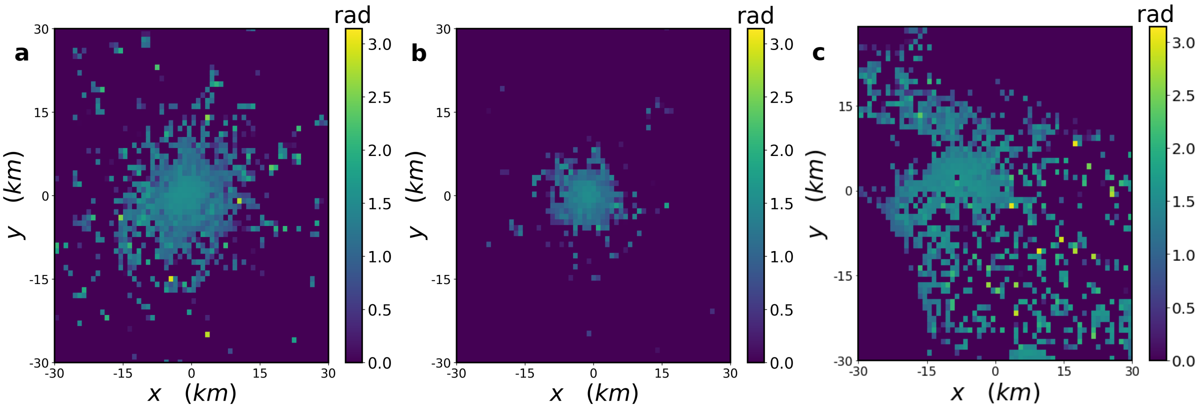

Supplementary Figure 48: **Angle Delta-map.** **a** London. **b** Paris. **c** Los Angeles.

We compare this empirical results with the same quantity calculated over a null model. To generate the null model we take all the outflows vectors we have in each cell and randomize their direction. We repeat this for 100 times.

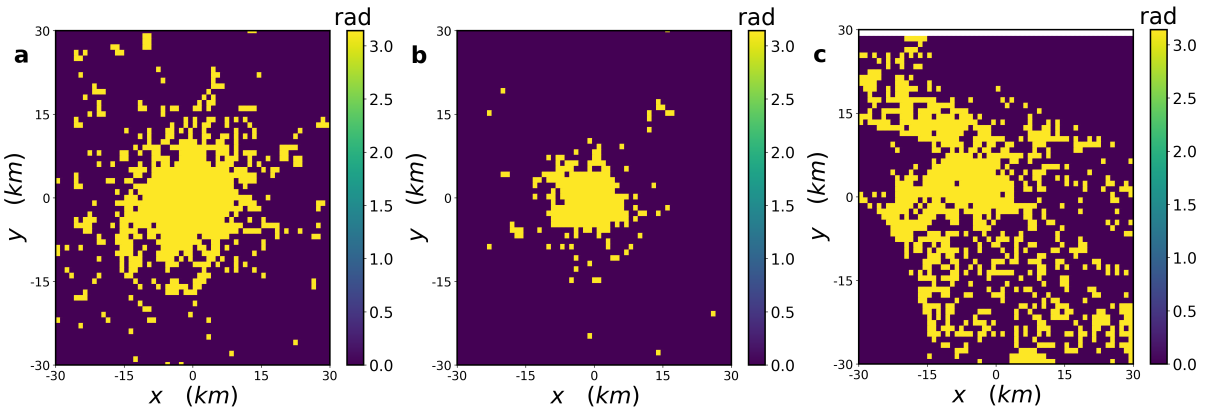

Supplementary Figure 49: **Angle null model Delta-map.** **a** London. **b** Paris. **c** Los Angeles.

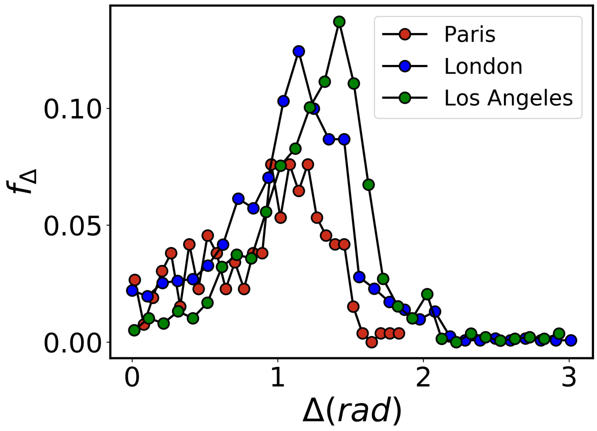

Supplementary Figure 50: Δ **distribution comparison** among **a** London. **b** Paris. **c** Los Angeles.

As shown in Fig.49, the null model gives results much wider than the empirical ones in each cell, i.e. the window of direction is fully open in the null model from $\Theta \in [-\pi, \pi]$.

To better understand the distribution of the empirical Δ, we plot the distribution for each city on a single histogram, as shown in Fig.50.

Supplementary Note 12: Collection of geolocated Twitter data

This code is an example in python of how to produce a query to the Twitter streaming API to obtain the results in a limited geographical BOX enclosed between latitude y0 and y1, and longitude x0 and x1. The aim of this code is only illustrative, the commands can be changed in any moment by the Twitter developers. An updated user guide on the usage of the Twitter streaming API can be found at `https://developer.twitter.com/en/docs`

```
from tweepy import Stream, OAuthHandler
from tweepy.streaming import StreamListener

CONSUMER_KEY = ''
CONSUMER_SECRET = ''
ACCESS_KEY = ''
ACCESS_SECRET = ''
BOX = [x0, y0, x1, y1]
class MyStreamListener(StreamListener):
  def on_status(self, status):
```

```
    print(status)

if __name__ == '__main__':
  auth = OAuthHandler(CONSUMER_KEY, CONSUMER_SECRET)
  auth.set_access_token(ACCESS_KEY, ACCESS_SECRET)
  listen = MyStreamListener()
  stream = Stream(auth, listen, gzip=True)
  stream.filter(locations=BOX)
```

Supplementary Note 13: An alternative definition of the home-work flows

Our treatment is symmetric to consider the commuting from home to work, as we do in the main paper, or from work to home. Summing up all the vector flows of commuters moving from work (cell j) to home (cell i) and assigning the resultant vector to work place, we get a vector $\vec{\mathbf{T}}_j^{\prime}$ in each cell j. This mesoscopic scale vector field describes a repulsive field, instead of an attractive field as the one in the main manuscript. As can be seen from Supplementary Figure 51, our findings are not altered by inverting the flows description. By comparing the potential generated by this empirical repulsive field with the originally defined attractive field, we see that the choice of defining commuting flows, from home to work or vice versa, does not alter the nature of the results regarding the potential and by extension neither those concerning the field.

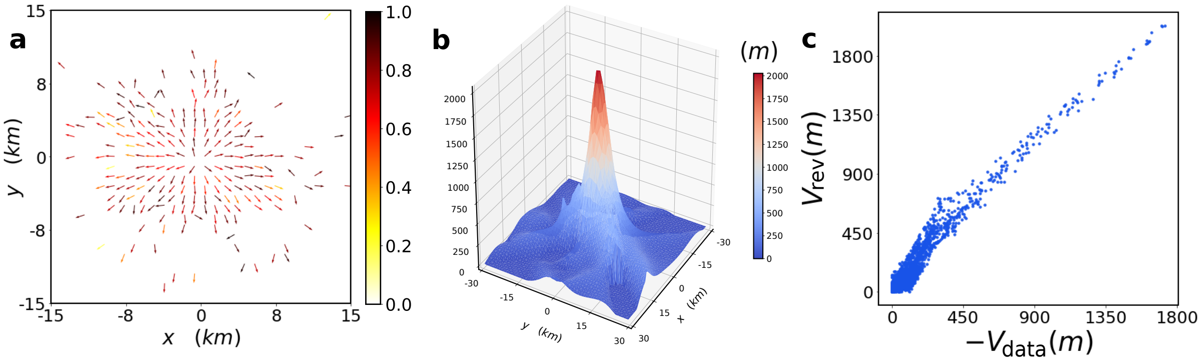

Supplementary Figure 51: **Inverting the flows in Paris.** **a** Vector field. **b** Potential. **c** Correlation plot between the original empirical potential and the repulsive field empirical potential (in opposite sign for the sake of comparison) $R_p^2 = 0.95$.

Supplementary References

[1] Boundaries of the UK 2011 census output areas. `https://geoportal.statistics.gov.uk/datasets/output-area-december-2011-full-extent-boundaries-in-england-and-wales/data`. Accessed: 2019-02-20.

[2] Yang, Y., Herrera, C., Eagle, N., and González, M. C. Limits of predictability in commuting flows in the absence of data for calibration. *Scientific Reports* **4**, 5662 (2014).

[3] Lenormand, M., Bassolas, A., and Ramasco, J. J. Systematic comparison of trip distribution laws and models. *Journal of Transport Geography* **51**, 158–169 (2016).
